# Bostrychines A–F, Six Novel Mycosporine-Like Amino-Acids and a Novel Betaine from the Red Alga *Bostrychia scorpioides*

**DOI:** 10.3390/md17060356

**Published:** 2019-06-14

**Authors:** Maria Orfanoudaki, Anja Hartmann, Helena Miladinovic, Hieu Nguyen Ngoc, Ulf Karsten, Markus Ganzera

**Affiliations:** 1 Institute of Pharmacy, Pharmacognosy, University of Innsbruck, Innrain 80−82, 6020 Innsbruck, Austria; Maria.Orfanoudaki@uibk.ac.at (M.O.); Helena.Miladinovic@student.uibk.ac.at (H.M.); hieu.nguyen-ngoc@student.uibk.ac.at (H.N.N.); markus.ganzera@uibk.ac.at (M.G.); 2 Institute of Biological Sciences, Applied Ecology & Phycology, University of Rostock, Albert-Einstein-Str. 3, 18059 Rostock, Germany; ulf.karsten@uni-rostock.de

**Keywords:** *Bostrychia scorpioides*, MAAs, Bostrychines A–F, betaines

## Abstract

Various red algae have repeatedly been reported to produce a variety of UV-absorbing mycosporine-like amino acids (MAAs), compounds that are well-known as natural sun-screens, as well as a plethora of betaines, metabolites which contribute to the osmotic balance under salt stress. Among other Rhodophyta, *Bostrychia scorpioides*, which is thriving as epiphyte on salt marsh plants in Europe and hence experiences extreme environmental conditions such as desiccation, UV-stress and osmotic stress, has barely been investigated for its secondary metabolites. In the present study, seven mycosporine like-amino acids and two betaines were isolated from *Bostrychia scorpioides* using various chromatographic techniques. Their structures were confirmed by Nuclear Magnetic Resonance (NMR) spectroscopy and High Resolution Mass Spectrometry (HRMS). Six MAAs and one betaine were chemically characterized as new natural products.

## 1. Introduction

The genus *Bostrychia* (Ceramiales, Rhodomelaceae) consists of approximately 40 species which are taxonomically accepted at present and they are widely distributed in tropical and warm temperate environments [1]. However, cryptic species are known to occur in several species complexes of *Bostrychia*, such as *B. tenella*, *B. radicans*, *B. moritziana*, *B. simpliciuscula*, *B. calliptera* and *B. intricata* [2,3]. Although the genus *Bostrychia* has recently received attention regarding its phylogeny and evolution, only a few studies have addressed their chemical composition, including reports on the occurrence of fatty acids, amino acids, carbohydrates, polysaccharides, betaines, phenolics, and mycosporine-like amino acids (MAAs) [4,5,6,7,8,9]. Regarding the latter, *Bostrychia* species have been reported to produce varying quantities of the UV-absorbing compounds mycosporine-glycine, shinorine, porphyra-334, palythine, asterina-330 and palythinol [9,10], however there are no detailed structural reports on the betaines.

The marine red alga *Bostrychia scorpioides* (Hudson) Montagne (Ceramiales, Rhodomelaceae) is the only species of this genus which abundantly grows on European coasts. Its habitat is above the high water tide mark on lower stems of saltmarsh flowering plants such as *Atriplex* (*Halimione*) *portulacoides*, *Spartina anglica*, *Pulcinella maritima* or *Sarcocornia perennis* [11,12,13]. *B. scorpioides* is wetted by sea water only during extreme high tides, and it is therefore able to survive several weeks exposed to air [12]. Consequently, Karsten et al. (1989) characterised *B. scorpioides* as a "terrestrial" red alga, due to its ability to tolerate incomplete turgor pressure regulation, which seems to be decisive for its uppermost intertidal distribution.

Growing under these conditions the alga experiences both severe osmotic and desiccation stresses. Therefore, it is interesting to understand its responses and osmotic acclimation during salinity stress. One of the protective mechanisms is the regulation of internal concentrations of the main ions K^+^, Na^+^, and Cl^−^ [12,14], and the concentration of organic compounds which are important osmolytes such as polyols, amino acids or betaines [12,15]. Like other species of the genus *Bostrychia*, *B. scorpioides* has evolved a CO_2_ photoassimilation process which is different from that of other red algae, by producing the isomeric hexitols, d-sorbitol, and d-dulcitol [16,17] instead of typical heterosides floridoside or digeneaside. Furthermore, *Bostrychia scorpioides* is reported to adjust its pigment content and photosynthetic performance, and is able to reduce its internal nitrogen and phosphor turnover at tidal levels allowing the species to cope with fluctuating environmental conditions [18].

Mycosporine-like amino acids form a compound class of water-soluble metabolites of low molecular weight with UV absorption maxima ranging from 268 to 360 nm [19,20,21]. They are distributed in a wide range of marine organisms [20,22], especially those inhabiting ecosystems that are highly insolated such as shallow marine environments, where MAAs are used as a natural UV-sunscreen against solar radiation [19]. The UV-absorbing properties of MAAs are well-established in the literature [19,21]. Numerous studies of MAAs from various organisms exposed to a high intensity of photosynthetically active radiation (PAR) and harmful ultraviolet radiation (UVR) suggest that MAA act as a protective sunscreen in marine organisms [21,22,23]. In addition, in recent years various MAAs are also considered as efficient antioxidants and heat dissipaters, and hence may also contribute to the desiccation tolerance and osmotic balance under salinity stress [20,22,24,25,26,27,28].

Betaines on the other hand, are zwitterionic quaternary amines with a widespread distribution in bacteria, fungi, animals, and plants [29]. In marine algae they occur in amounts of less than 1% (dry weight), and their yields are generally higher in Rhodophyta and Chlorophyta compared to brown algae [30]. The most common betaines in algae are glycine-betaine, γ-aminobutyric acid-betaine, proline-betaine, and 3-dimethylsulphoniopropionate. Their role as osmoprotectants is well-established since they are known to contribute to survival and cellular adaptation under osmotic pressure and they can have taxonomic significance at the generic level [30,31,32].

The focus of the present study was to investigate the chemical composition of *B. scorpioides* targeting stress metabolites like MAAs and betaines since its saltmarsh habitat is unique and quite different from those of other *Bostrychia* species and other red algae. Thus, the extreme environment may affect the biosynthesis and biochemistry of this species, and lead to the production of yet unexplored molecules. To date there is only one study from Karsten et al. (2000), regarding the presence of MAAs in *B. scorpioides*. These authors reported that a field sample of *B. scorpioides* from the Netherlands contained an unknown MAA together with shinorine, palythine, and asterina-330 whereas a field sample from France contained mycosporine-glycine, shinorine, palythine, and asterina-330 [10].

## 2. Results

**Figure 1:**
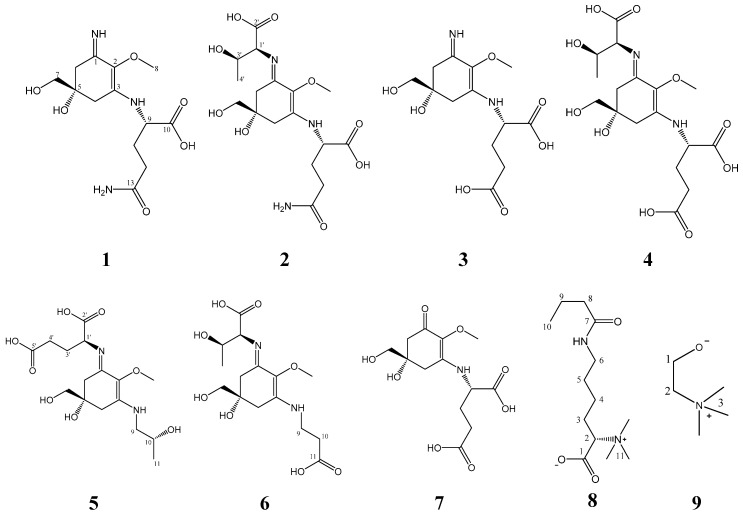
The chemical structures of compounds **1**–**9** as elucidated by Nuclear Magnetic Resonance (NMR) spectroscopy.

The methanolic extract of *B. scorpioides* was fractionated on a silica gel column, followed by several separation steps using flash chromatography on reversed phase material (C-18). After final purification on a semi-preparative High-Performance-Liquid-Chromatography (HPLC), nine compounds (Figure 1) were isolated.

### 2.1. Compound **1**

**Figure 2:**
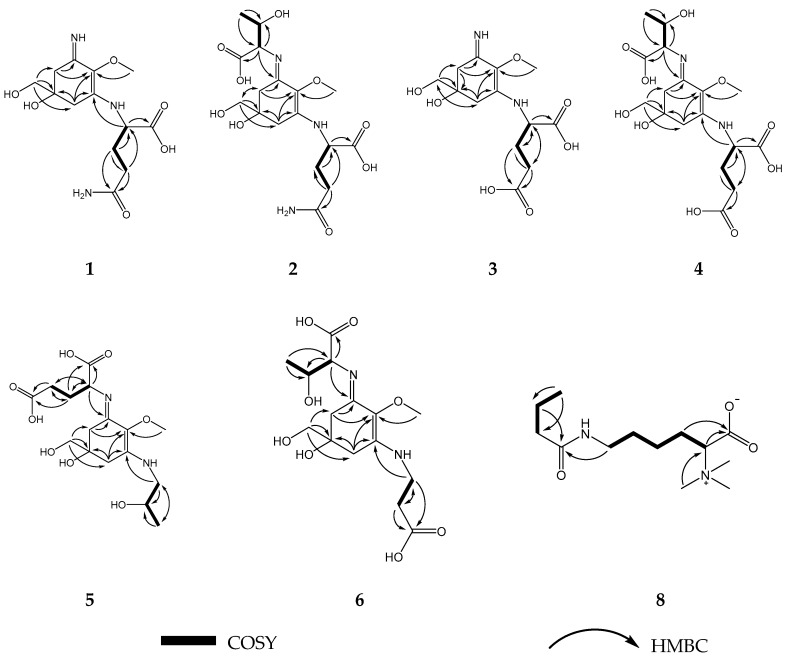
Key HMBC (^1^H → ^13^C) and ^1^H-^1^H COSY correlations of compounds **1**–**6** and **8**.

Characteristic Nuclear Magnetic Resonance (NMR) shifts indicated the presence of a cyclohexenimine-type MAA scaffold. The side chain was identified as glutamine based on a COSY correlation of H-9/H-11/H-12 and two carbonyl groups at *δ*_C_ 179.1 and 181.1, and by comparison with literature values [33]. Its position was confirmed by long-range correlations visible in the HMBC spectrum (H-9 at *δ*_H_ 4.22 to C-3 at *δ*_C_ 163.9), and relevant connectivities are indicated by arrows in Figure 2. Specifically, a methine at *δ*_H_ 4.22 (H-9) showed a correlation in the COSY spectrum with the protons of the methylene at *δ*_H_ 2.18 and *δ*_H_ 2.26 (H-11), and an HMBC correlation with the carbonyl group at *δ*_C_ 179.1 (C-10). Furthermore, the protons of the methylene at C-12 (*δ*_H_ 2.45) showed a correlation in the COSY spectrum with the protons of the methylene at position 11 (*δ*_H_ 2.18 and 2.26) and an HMBC correlation with the carbonyl group at *δ*_C_ 181.1 (C-13). Glutamine has already been found individually in another MAA, mycosporine-glutamine. The latter was discovered by Bernillon et al., in 1983 [34]. Advanced Marfey’s method confirmed the presence of l-glutamic acid as final reaction product (Figure S8) indicating l-configuration as absolute configuration of the glutamine at the MAA. Glutamine is reported to be instable during the incubation with hydrochloric acid at 100 °C resulting in the degradation products pyroglutamic acid and glutamic acid [35]. Since pyroglutamic acid is a secondary amine, it will not react with Marfey’s reagent in contrast to glutamic acid which is a primary amine. As for the chiral carbon 5, it is depicted in all MAAs of the present study with the generally accepted configuration [36,37,38,39,40] although further studies have to prove the absolute configuration of this stereochemical center. Compound **1** was finally identified as a new MAA, Palythine-Glutamine or ((*S*)-5-hydroxy-5-(hydroxymethyl)-3-imino-2-methoxycyclohex-1-en-1-yl)-l-glutamine, with the molecular formula: C_13_H_21_N_3_O_6_ (high resolution mass spectrometry data: [M + H]^+^ = 316.1472, [2M + Na]^+^ = 653.2617, Figure S7), it was given the trivial name bostrychine-A.

However, the ^1^H-NMR spectrum of compound **1** showed three additional signals corresponding to a known betaine, choline as an impurity (compound **9**, ratio 3:1 as established by the integration in the ^1^H-NMR spectrum). The occurrence of choline was determined by the presence of a methylene at *δ*_H_ 3.52 (*δ*_C_ 70.2) which is visible in a COSY correlation with the protons of a second methylene at *δ*_H_ 4.06 and an HMBC correlation with three methyl groups at *δ*_C_ 58.5 (*δ*_H_ 3.20, 9H). Choline was additionally confirmed by LC-MS analysis since the respective chromatogram showed two main peaks (Figure S9). The first peak is corresponding to choline (chemical formula: C_5_H_13_NO), with MS values of *m*/*z* 104.3 [M + H]^+^ and *m*/*z* 207.3 [2M + H]^+^. The second peak is corresponding to compound **1** with an *m*/*z* value of 316.2 [M + H]^+^.

### 2.2. Compound **2**

Characteristic NMR shifts and 2D-NMR data (Table 1 and Table 2) of compound **2** indicated the same substructure as compound **1**. However, the ^1^H-NMR spectrum showed three extra signals, two doublets at *δ*_H_ 4.07 (*J* 4.7 Hz, H-1′) and *δ*_H_ 1.25 (*J* = 6.6 Hz, H-4′) and a multiplet at *δ*_H_ 4.31 (H-3′). The protons of the methine at *δ*_H_ 4.07 (H-1′) showed correlation in the HMBC spectrum with the carbon at *δ*_C_ 178.1 (C-2′) revealing the presence of a threonine moiety. The HMBC correlation of the same proton (H-1′) to carbon C-1 at *δ*_C_ 162.4 indicated that threonine is attached to position C-1. The presence of l-glutamic acid and l-threonine as constituents was again confirmed by LC-MS using the advanced Marfey’s method (Figure S16). As the NMR data of all substructures were in good agreement with literature values, compound **2** was finally identified as a new MAA, mycosporine-threonine-glutamine or ((Bostrychine)-3-(((1*S*,2*R*)-1-carboxy-2-hydroxypropyl)imino)-5-hydroxy-5-(hydroxymethyl)-2-methoxycyclohex-1-en-1-yl)-l-glutamine with molecular formula: C_17_H_27_N_3_O_9_ (high resolution MS data: [M + H]^+^ = 418.1739, Figure S15), for which we propose the trivial name bostrychine-B.

### 2.3. Compound **3**

Analyses of the 2D-NMR spectra of compound **3** revealed a high similarity to compound **1**. However, carbons 12 and 13 were slightly deshielded (Table 1 and Table 2) indicating the presence of a glutamic acid moiety instead of glutamine. This was supported by high resolution MS data corresponding to [M + H]^+^ = 317.1310 (Figure S22), from which the molecular formula C_13_H_21_N_3_O_6_ was established. Glutamic acid has been reported twice in the literature as a substituent at the cyclohexenimine MAAs i.e., mycosporine-glycine-glutamic acid and mycosporine-glutamic acid [41]. NMR data of both substructures were in good agreement with literature values and advanced Marfey’s method showed that the absolute configuration of glutamic acid in the MAA was L (Figure S23). Thus, compound **3**, named bostrychine-C, was identified as palythine-glutamic acid or ((*S*)-5-hydroxy-5-(hydroxymethyl)-3-imino-2-methoxycyclohex-1-en-1-yl)-l-glutamic acid, a new MAA with the molecular formula: C_13_H_20_N_2_O_7_.

### 2.4. Compound **4**

2D-NMR spectra of compound **4** were highly similar to those of compound **2** revealing a cyclohexenimine scaffold and a threonine moiety. Additionally, high resolution MS data were recorded, corresponding to [M + H]^+^ = 419.1172 and [M − H]^−^ = 417.1469 (Figure S29) from which the molecular formula C_17_H_26_N_2_O_10_ could be established. However, carbons 12 and 13 of compound **4** differed slightly from those of compound **2**, and characteristic NMR shifts and 2D-NMR spectra (Table 1 and Table 2) confirmed the presence of a glutamic acid moiety attached to carbon atom 3 (*δ*_C_ 163.4) of the cyclohexenimine skeleton. The absolute configuration of both amino acids was confirmed to be *L* (Figure S30). Thus, compound **4**, bostrychine-D with molecular formula: C_17_H_26_N_2_O_10_, was identified as mycosporine-threonine-glutamic acid or ((*S*,*E*)-3-(((1*S*,2*R*)-1-carboxy-2-hydroxypropyl)imino)-5-hydroxy-5-(hydroxymethyl)-2-methoxycyclohex-1-en-1-yl)-l-glutamic acid.

### 2.5. Compound **5**

Comparison of the 2D-NMR spectra of compound **5** to that of compound **3** indicated a high degree of similarity. However, in the ^1^H-NMR spectrum four additional signals were noticed, two doublets at *δ*_H_ 3.44 and 3.51 (H-9), a multiplet at *δ*_H_ 4.02 (H-10), and a doublet at *δ*_H_ 1.22 (H-11). Further analyses of the 2D-NMR spectra (Table 1 and Table 2) of compound **5** indicated the presence of a threamine (1-amino-2-propanol) moiety. Threamine has been reported in the literature as a substitution of the cyclohexenimine skeleton already, for example in the MAA aplysiapalythine A [42].

The HMBC correlation of protons H-9 of the threamine moiety (*δ*_H_ 3.44 and 3.51) to carbon C-3 at *δ*_C_ 164.5 and of proton H-1’ of the glutamic acid moiety (*δ*_H_ 4.43) to carbon C-1 at 1 *δ*_C_ 61.8 indicated at which position, threamine and glutamic acid are attached to the cyclohexenimine scaffold. Their absolute configuration l-glutamic acid *R*-threamine was confirmed as described before (Figure S38 and S39). Since the NMR data of all substructures were in good agreement to literature values, compound **5** was finally identified as a new MAA, mycosporine-threamine-glutamic acid or (*S*)-2-(((*S*,*E*)-5-hydroxy-5-(hydroxymethyl)-3-(((*R*)-2-hydroxypropyl)amino)-2-methoxycyclohex-2-en-1-ylidene)amino)pentanedioic acid. The compound was named bostrychine-E, and it has the molecular formula C_16_H_26_N_2_O_8_ (high resolution MS data: [M + H]^+^ = 375.1737, Figure S37).

### 2.6. Compound **6**

Based on the characteristic NMR shifts (Table 1 and Table 2) of compound **6** a cyclohexenimine-scaffold and the presence of the amino acids threonine and β-alanine could be concluded. The HMBC correlation of proton H-9 of the β-alanine moiety (*δ*_H_ 3.76) to carbon C-3 at *δ*_C_ 164.2 indicated that β-alanine is attached to carbon atom 3, whereas an HMBC correlation of proton H-1′ of the threonine moiety (*δ*_H_ 4.35) to carbon C-1 at *δ*_C_ 161.8 confirmed that threonine is attached at this position to the cyclohexenimine scaffold. By LC-MS the absolute configuration of glutamic acid could be determined to be L (Figure S47). As the NMR data of all substructures were in good agreement to literature values, compound **6** was finally identified as a new MAA, mycosporine-threonine-β-alanine or (2*S*,3*R*)-2-(((*S*,*E*)-3-((2-carboxyethyl)amino)-5-hydroxy-5-(hydroxymethyl)-2-methoxycyclohex-2-en-1-ylidene)amino)-3-hydroxybutanoic acid. Its molecular formula is C_15_H_24_N_2_O_8_ (high resolution MS data: [M + H]^+^ = 361.1586, Figure S46) and we suggest the name bostrychine-F for this novel natural product.

### 2.7. Compound **7**

Characteristic NMR shift values (Section 4.4.7) of compound **7** indicated an MAA with cyclohexenone-scaffold and the presence of glutamic acid. As NMR data were in good agreement with literature values [40], compound **7** was identified as the known MAA mycosporine-glutamic acid. Its molecular formula is C_13_H_19_NO_8_ (ESIMS *m*/*z* 318 [M + H]^+^; ESIMS *m*/*z* 316 [M − H]^−^).

### 2.8. Compound **8**

The ^1^H-NMR spectrum of compound **8** revealed the presence of five methylene groups, a triplet-type methyl group at *δ*_H_ 0.90 (3H, s), three methyl groups observed as a singlet at *δ*_H_ 3.18 (9H, s), as well as a methine group at *δ*_H_ 3.61. The ^13^C NMR spectra showed the appearance of two carbonyl groups at *δ*_C_ 174.8 and 180.0. The COSY spectrum revealed two coupling networks, including H-2/H-3/H-4/H-5/H-6 and H-8/H-9/H-10. Two substructures were connected through an amide bond by specific chemical shifts of position 6 (*δ*_H_ 3.21, *δ*_C_ 41.5) and 8 (*δ*_H_ 2.21, *δ*_C_ 40.6), and key HMBC correlations of H-6, 8, 9/C-7 (*δ*_C_ 180.0). Furthermore, the HMBC correlations of H-2 (*δ*_H_ 3.61) and H-3 (*δ*_H_ 1.86 and 1.95) to C-1 (*δ*_C_ 174.8), as well as those of the methyl groups at *δ*_H_ 3.18 (H-11) to C-2 (*δ*_C_ 81.8) confirmed the presence of a betaine group. Compound **8** was assigned the molecular formula C_13_H_26_N_2_O_3_ as established by positive ions [M + Na]^+^ at *m*/*z* 281.1809 and [2M + Na]^+^ at *m*/*z* 539.3740 (Figure S53). Collectively, compound **8** was finally identified as (*S*)-6-butyramido-2-(trimethylammonio)hexanoate or butyryl-lysine-betaine, a new natural product.

**Figure 3:**
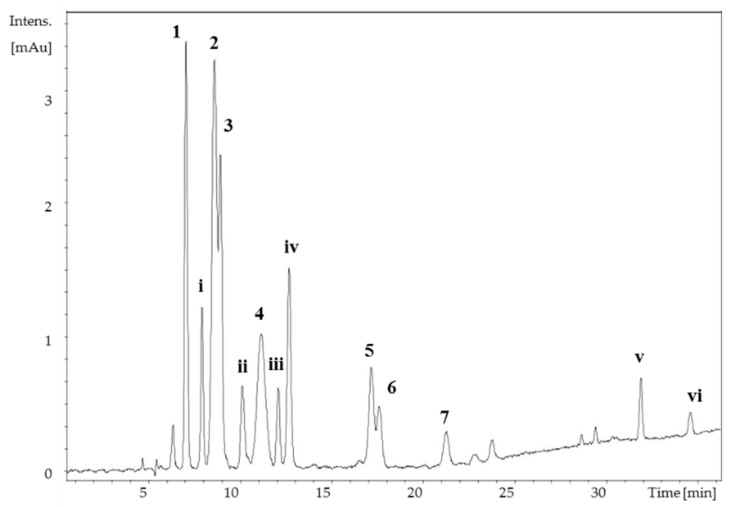
HPLC-UV separation of the *Bostrychia scorpioides* MeOH extract. Peak assignment is according to Figure 1. **1**–**7**: isolated mycosporine-like amino acids (MAAs), **i**–**vi**: unidentified compounds (Table S1), column: YMC-Pack ODS (250 × 4.60 mm, 5 μm) mobile phase: 20 mM ammonium formate and 0.6% (*v*/*v*) formic acid in water (A) and methanol (B); gradient: 0–15 min: 2% B, 23 min: 10% B, 30 min: 15% B, 35–40 min: 98% B, 40.1–50 min: 2% B; λ = 310, 330, and 350 nm; flow rate = 0.6 mL/min; T = 20 °C.

Apart from the isolated compounds, six additional MAAs were present in the methanolic extract of *B. scorpioides*. Due to the minor concentration of compounds **i**, **ii** in the extract (Figure 3), their isolation was not feasible. Compounds **iii**–**vi** on the other hand were instable, and despite the higher concentration of some of them in the extract, their isolation and structure elucidation was also impossible. Their absorption maxima and molecular masses are summarized in Table S1.

## 3. Discussion and Conclusions

The research on mycosporine-like amino acids, molecules well-known for their UV-protective properties, has gained the attention of the scientific community and cosmetic industry during the last decades not only because they can act as sunscreens in marine organisms [21,22,23] but also as efficient antioxidants and multifunctional secondary metabolites [22].

*B. scorpioides* was selected for this study because of its unique and extreme natural habitat, as well as due to an interesting MAA pattern, during an initial HPLC screening of the extract. Subsequently, six novel MAAs, mycosporine-glutamic acid and one novel betaine were isolated using a purification protocol which included silica gel chromatography, flash chromatography and semi-preparative HPLC. All novel MAAs had a cyclohexenimine-scaffold with the amino acids, glutamic acid, glutamine and threonine as side chains, while the betaine was a lysine derivative. The MAA pattern of this species is therefore completely different to that of other species from the genus *Bostrychia* or other red algae which usually contain porphyra-334, shinorine, asterina-330, palythine, aplysiapalythine-A, mycosporine-glycine, aplysiapalythine B, mycosporine-glycine-alanine, mycosporine-methylamine-threonine, palythene and usujirene [43].

Additionally, six further compounds (compounds **i**–**vi**) were detected in the extract of *B. scorpioides*, but because of either low concentration or poor stability, their structure could not be elucidated. However, the absorption maxima and molecular masses of compounds **ii** and **iii** would agree with those of mycosporine-glycine-aspartic acid, isolated previously from a brine shrimp [44] and mycosporine-glutamine, isolated from a fungus [35], respectively. The presence of mycosporine-glutamine in this alga would be plausible, given the presence of mycosporine-glutamic acid, palythine- glutamine and mycosporine-threonine-glutamine (compounds **1**–**3**), as well as its instable nature, which is characteristic for cyclohexenone type MAAs. Additionally, compounds **v** and **vi** showed absorption maxima in the range of 360 nm, which is unique because only two other MAAs have been reported to exhibit so high absorption maxima (357–360 nm), namely usujirene and palythene. However, due to their high instability the structure of compounds **v** and **vi** could not be elucidated. The absorption maxima and molecular masses of compounds **i**–**vi** are summarized in Table S1.

The present data is in contrast with the results published by Karsten et al. in 2000 [10], which reported that samples of *Bostrychia scorpioides* contained an MAA present also in *Catenella* sp., shinorine, palythine, asterina-330, and mycosporine-glycine. However, compared to our approach this earlier publication was only based on a HPLC method without coupling to an MS detector for a deeper chemical characterization. In addition, it might be possible that samples of *Bostrychia* from different biogeographical origins produce different MAAs, since we know from other taxa that biogeography is an important factor for shaping chemical profiles in red algae due to variabilities in environmental settings (e.g., solar radiation, day length, temperature, salinity, nutrients etc.) [45].

In the salt marsh habitat *B. scorpioides* is exposed to higher environmental irradiance levels than other *Bostrychia* species in tropical habitats, where they preferentially grow as epiphytes on the prop roots of mangroves which provide shade by the canopy [10,46,47]. The biochemical capability to synthesize and accumulate MAAs is a highly efficient photoprotective mechanism. While many intertidal red algae typically contain three to five different MAAs for photoprotection, the relatively high number of 12 compounds in *B. scorpioides* is rather unusual and might be explained by the rather unique habitat. In our recent study *Bostrychia arbuscula* exhibited only five MAAs [43]. These compounds act as passive shielding solutes by dissipating the absorbed solar UV radiation energy in the harmless form of heat without generating photochemical reactions [48]. MAAs are chemically characterized by extremely high molar absorptivity for UVA and UVB (molar extinction coefficients between 28.000 and 50.000), and have been reported to be photochemically stable molecules, both of which are prerequisites for their experimentally proven sunscreen function [49].

To date, the role of MAAs for maintaining osmotic homeostasis is not very clear. Many studies report that dilution stress leads to an immediate excretion of MAAs from the cells or that the MAA content increases with a rise in salinity [50,51,52]. On the other hand, the MAA concentrations that are reported, although being significant, are still much lower than those required to balance the salinity stress [27]. Therefore, these substances might act as supplementary osmolytes together with other osmotic solutes like betaines or low molecular weight carbohydrates such as floridoside or polyols [26,51]. Taking into consideration that *B. scorpioides* grows in salt marshes on herbs and scrubs where it is regularly confronted with osmotic stress, it would be interesting to investigate whether the isolated MAAs are linked to increases and decreases in external salinity. On the other hand, the osmoprotective role of betaines in organisms which experience severe osmotic and desiccation stress is well-documented [30,53] and together with low molecular weight carbohydrates they are considered the main metabolites contributing to osmotic regulation. Therefore, it is reasonable to assume such an ecophysiological role of the novel betaine and choline found in *B. scorpioides*. Together with digeneaside, d-sorbitol, and d-dulcitol this species contains an array of organic osmolytes which contribute to the high salinity tolerance of this red alga and thus explain the ecological success under the extreme and fluctuating environmental conditions in salt marshes.

## 4. Materials and Methods

### 4.1. Biological Material

The algal material was collected at Plouescat, France on 14 June 2018 and was morphologically identified by the author U. Karsten using his taxonomic expert knowledge in conjunction with standard identification keys [1,54]; the alga was air-dried and stored at 22 °C until further processing. A voucher sample is deposited at the Institute of Pharmacy, Pharmacognosy, University of Innsbruck, Austria.

### 4.2. Instrumentation

NMR experiments were conducted on a Bruker Avance II 600 spectrometer (Karlsruhe, Germany) operating at 600.19 (^1^H) and 150.91 MHz (^13^C). The isolated compounds were dissolved in deuterated water from Euriso-Top (Saint Aubin, France) using tetramethylsilane (TMS) as internal standard. High-resolution mass spectra were measured with a micrOTOF-Q II mass spectrometer (Bruker-Daltonics, Bremen, Germany). The experiments were performed in the positive or negative ESI mode with the following parameters: capillary energy, 4500 V for positive mode and 3500 V for negative mode; nebulizer gas, 6.4 psi; dry gas, 4.0 L/min at a temperature of 180 °C; the recorded scan range was 100–600 *m*/*z*. Low-resolution mass spectra were measured with an Agilent InfinityLab LC/MSD System comprising of an Agilent HPLC 1260 HPLC, equipped with a binary pump, autosampler, column oven and photodiode array detector Agilent 1260 HPLC (Santa Clara, CA, USA). The experiments were performed in the positive or negative ESI mode with the following parameters: capillary energy, 4000 V; nebulizer gas, 40.0 psi; dry gas, 10.0 L/min at a temperature of 300 °C; the recorded, scan range was 100–1500 *m*/*z*.

For the purification of compounds, a Reveleris^®^ X2 iES flash chromatography system (Büchi, Flawil, Switzerland) and a semi-preparative Dionex UltiMate 3000 HPLC (Thermo, Waltham, MA, USA), comprising a P580 pump, an ASI 100 auto-mated sample injector, an UVD 170 U detector and a fraction collector, were used. Analytical HPLC experiments were performed on a Shimadzu LC-20AD XR system. Optical rotations were measured using a Jasco P-2000 digital polarimeter (Jasco Austria, Biolab GmbH and Co., Wien, Austria).

### 4.3. Chemicals and Reagents

All solvents required for extraction and isolation were purchased from VWR International (Vienna, Austria), and ethyl acetate was distilled before use. Solvents for analytical experiments had at least pro analysis (p.a.) quality and were obtained from Merck (Darmstadt, Germany). Deuterated solvents were supplied by Euriso-Top (Saint-Aubin Cedex, France). Ultrapure water was produced by a Sartorius arium^®^ 611 UV (Göttingen, Germany) purification system. Silica gel 40–63 μm and pre-packed cartridges for flash chromatography were purchased from Merck (Darmstadt, Germany) and Büchi (Flawil, Switzerland), respectively.

### 4.4. Extraction and Isolation

The plant material (990 g) was crushed to powder in a grinding mill and extracted thrice in an ultrasonic bath (Bandelin Sonorex 35 KHz, Berlin, Germany) for 15 min using dichloromethane (10 L in total) which afforded 3.7 g of dichloromethane extract (yield 0.4%). For MAA extraction the remaining dry plant material was first extracted using pure methanol (approximately 20 L) under the same conditions (73 g of methanolic extract, yield 7.4%), followed by a threefold extraction with methanol/water = 1/1 (*v*/*v*) (4 L of solvent) which afforded 58 g of water extract (yield 5.9%). HPLC analysis of the extracts indicated that the methanol extract contained MAAs, therefore it was selected for further fractionation.

The methanolic extract (73 g) was fractionated on a silica gel column (80 × 5 cm, 800 g of silica gel) using gradient elution (EtOAc to methanol), resulting in 21 fractions. Fractions 15, 16, and 17 were individually fractionated with flash chromatography, using a C-18 40 g cartridge from Büchi. Elution was carried out by the following water (A) and MeOH (B) gradient: 0–15 min: 0% B, 25 min: 100% B, 25–40 min: 100% B. The flow rate was 10 mL min^−1^ and UV detection performed at 254, 310, and 350 nm.

Flash chromatography of fraction 15 (3.5 g) resulted in six sub-fractions. Sub-fraction 15b (2.3 g) was subjected to semi-preparative HPLC using a Synergi 4 u Polar-RP column (250 × 10 mm, 4 µm; Phenomenex, Torrance, CA, USA). The mobile phase comprised of 0.1% (*v*/*v*) trifluoroacetic acid in water (A) and 0.25% (*v*/*v*) formic acid in methanol (B) and the following gradient was used: 0 min: 2% B, 15 min: 2% B, 20.1 min: 50% B, 25 min: 98 % B, 30 min: 98% B, 30.1–40 min: 2% B. The separation was monitored at 330 nm, column temperature and flow rate were set to 21 °C and 1.3 mL/min, respectively. This resulted in the isolation of compounds **2** (1.5 mg) and **3** (0.96 mg). Sub-fraction 15e (170 mg) was subjected to semi-preparative HPLC on an Aqua C18 column (250 × 10 mm, 5 μm; Phenomenex), using a mobile phase comprising of 0.25% (*v*/*v*) formic acid in water (A) and methanol (B); the applied gradient was: 0 min: 2% B, 5 min: 5% B, 25 min: 10% B, 25.1–30 min: 2% B. The separation was monitored at 330 nm, column temperature and flow rate were set to 21 °C and 1.3 mL/min, respectively. This step resulted in the isolation of compounds **1** and **9** as a mixture (6.9 mg).

Flash chromatography of fraction 16 (2.6 g) resulted in seven subfractions. Sub-fraction 16b (1 g) was subjected to semi-preparative HPLC using a Synergi 4 u Polar-RP column (250 × 10 mm, 4 µm; Phenomenex, Torrance, CA, USA). The mobile phase comprised of 0.1% (*v*/*v*) trifluoroacetic acid in water (A) and 0.25% (*v*/*v*) formic acid in methanol (B) and the following gradient was used: 0 min: 2% B, 5 min: 10% B, 25 min: 20% B, 30 min: 20% B, 35 min: 40% B, 40 min: 98% B, 40.1 min: 2% B, 55 min: 2% B. The separation was monitored at 355 nm, column temperature and flow rate were set to 21 °C and 1.3 mL/min, respectively. The goal of this isolation step was the purification of compounds **v** and **vi** which were unstable and therefore finally could not be identified. However, this purification step led to the isolation of compound **8** (3.1 mg) whose presence was confirmed by LC-MS analysis. Sub-fraction 16c (71 mg) was subjected to semi-preparative HPLC using the same column and solvent system (UV detection wavelength: 330 nm, column temperature: 21 °C and flow rate: 1.3 mL/min). The following gradient was used: 0 min: 2% B, 55 min: 2% B, resulting in the isolation of compounds **5** (3.9 mg) and **6** (6.6 mg).

Flash chromatography of fraction 17 (2.7 g) resulted in six subfractions. Sub-fraction 17b (2.3 g) was subjected to semi-preparative HPLC using a Synergi 4 u Polar-RP column (250 × 10 mm, 4 µm; Phenomenex, Torrance, CA, USA). The mobile phase comprised of 0.25% (*v*/*v*) formic acid in water (A) and methanol (B) and the following gradient was used: 0 min: 2% B, 5 min: 5% B, 25 min: 10 % B, 25.1–35 min: 2% B (UV detection wavelength: 330 nm, column temperature: 21 °C and flow rate: 1.3 mL/min). This resulted in the isolation of compounds **4** (5.9 mg) and **7** (0.8 mg).

#### 4.4.1. Bostrychine-A (**1**)

White amorphous powder; UV λmax = 322 nm; ^1^H and ^13^C NMR data (400/100 MHz; D_2_O), Table 1 and Table 2; ESIMS *m*/*z* 316 [M + H]^+^; HRESIMS *m*/*z* 316.1472 [M + H]^+^ (calcd. for C_13_H_22_N_3_O_6_, 316.1509).

#### 4.4.2. Bostrychine-B (**2**)

Pale yellow amorphous powder; [α]^21^_D_ = −29.1 (c 1, H_2_O); UV λmax = 335 nm; ε = 36,155 M^−1^·cm^−1^; ^1^H and ^13^C NMR data (600/150 MHz; D_2_O), Table 1 and Table 2; ESIMS *m*/*z* 418 [M + H]^+^; HRESIMS *m*/*z* 418.1739 [M + H]^+^ (calcd. for C_17_H_28_N_3_O_9_, 418.1825).

#### 4.4.3. Bostrychine-C (**3**)

White amorphous powder; [α]^21^_D_ = –33.3 (c 1, H_2_O); UV λmax = 322 nm; ε = 22,351 M^−1^·cm^−1^; ^1^H and ^13^C NMR data (600/150 MHz; D_2_O), Table 1 and Table 2; ESIMS *m*/*z* 317 [M + H]^+^; HRESIMS *m*/*z* 317.1310 [M + H]^+^ (calcd. for C_13_H_21_N_2_O_7_, 317.1348).

#### 4.4.4. Bostrychine-D (**4**)

Pale yellow amorphous powder; [α]^21^_D_ = –1.9 (c 0.1, H_2_O); UV λmax = 337 nm; ε = 31,956 M^−1^·cm^−1^; ^1^H and ^13^C NMR data (600/150 MHz; D_2_O), Table 1 and Table 2; ESIMS *m*/*z* 419 [M + H]^+^; HRESIMS *m*/*z* 419.1172 [M + H]^+^ (calcd. for C_17_H_25_N_2_O_10_, 419.1666); and *m*/*z* 417.1469 [M − H]^−^ (calcd. for C_17_H_25_N_2_O_10_, 417.1509).

#### 4.4.5. Bostrychine-E (**5**)

Pale yellow amorphous powder; [α]^21^_D_ = –2.2 (c 0.1, H_2_O); UV λmax = 333 nm; ε = 21,618 M^−1^·cm^−1^; ^1^H and ^13^C NMR data (600/150 MHz; D_2_O), Table 1 and Table 2; ESIMS *m*/*z* 375 [M + H]^+^; HRESIMS *m*/*z* 375.1737 [M + H]^+^ (calcd. for C_16_H_27_N_2_O_8_, 375.1767).

#### 4.4.6. Bostrychine-F (**6**)

Pale yellow amorphous powder; [α]^21^_D_ = –2.4 (c 0.1, H_2_O); UV λmax = 332 nm; ε = 44,994 M^−1^·cm^−1^; ^1^H and ^13^C NMR data (600/150 MHz; D_2_O), Table 1 and Table 2; ESIMS *m*/*z* 361 [M + H]^+^; HRESIMS *m*/*z* 361.1586 [M + H]^+^ (calcd. for C_15_H_25_N_2_O_8_, 361.1611).

#### 4.4.7. Mycosporine-Glutamic Acid (**7**)

White amorphous powder; UV λmax = 310 nm; ^1^H NMR data (600 MHz; D_2_O), *δ*_H_ 4.12 (1H, m, H-9); 3.59 (3H, s, H-8); 3.52 (2H, s, H-7); 2.75 (1H, d, *J* = 17.3, Hz H-6); 2.69 (1H, d, *J* = 17.3 Hz, H-6); 2.67 (1H, d, *J* = 17.1 Hz, H-4); 2.39 (1H, d, *J* = 17.1 Hz, H-4); 2.49 (2H, t, *J* = 7.4 Hz, H-12); 2.20 (1H, m, H-11); 2.10 (1H, m, H-11), ESIMS *m*/*z* 318 [M + H]^+^; ESIMS *m*/*z* 316 [M − H]^−^.

#### 4.4.8. Butyryl-lysine-betaine (**8**)

Pale yellow amorphous powder; [α]^21^_D_ = +5.1 (c 0.1, H_2_O); ^1^H NMR data (600 MHz; D_2_O), *δ*_H_ 3.61 (1H, dd, *J* = 11.8/3.4, H-2); 3.22 (2H, m, H-6); 3.18 (3H, s, H-11); 2.21 (2H, t, *J* = 7.4, H-8); 1.95 (1H, m, H-3); 1.90 (3H, t, *J* = 7.4, H-10); 1.86 (1H, m, H-3); 1.60 (2H, m, H-5); 1.60 (2H, m, H-9); 1.37 (2H, m, H-4); ^13^C NMR data (150 MHz; D_2_O), Table 2; ESIMS *m*/*z* 258 [M + H]^+^; HRESIMS *m*/*z* 281.1181 [M + Na]^+^ (calcd. for C_13_H_26_N_2_O_3_Na, 281.1841), and *m*/*z* 539.3740 [2M + Na]^+^ (calcd. for C_26_H_52_N_4_O_6_Na, 539.3784).

#### 4.4.9. Choline (**9**)

White amorphous powder; ^1^H NMR data (400 MHz; D_2_O), *δ*_H_ 4.06 (2H, m, H-1); 3.52 (2H, m, H-2); 3.20 (9H, s, H-3); ^13^C NMR data (100 MHz; D_2_O), *δ*_C_ 70.2 (C-2); 58.5 (C-3); 56.8 (C-1), ESIMS *m*/*z* 104.3 [M + H]^+^ and at *m*/*z* 207.3 [2M + H]^+^.

### 4.5. Determination of the Absolute Configurations of Amino Acids in MAAs by the Advanced Marfey’s Method Using LC-MS

The general procedure was adapted from a previously published protocol [55]. Approximately 0.1 mg of each MAA was stirred with HCl 37% (250 μL) at 90 °C for 120 min. The hydrolysates were evaporated to dryness and then resuspended in H_2_O (100 μL).

Each amino acid (200 μg) or hydrolysate (ca. 100 µg) was dissolved in 1 M NaHCO3 (200 μL) and 1% d- or l-1-fluoro-2,4-dinitrophenyl-5-l-leucinamide (FDLA) derivatization reagent in acetone (25 μL) was added. The reaction vials were incubated and stirred for 30 min at 50 °C. The reactions were then quenched with 2 N HCl (100 μL). MeOH (800 μL) was added to prepare LC-MS samples. The reaction products were analyzed by HPLC-MS, using a Luna 5µ C-8 column (150 × 4.6 mm, 5 µm; Phenomenex, Torrance, CA, USA). H_2_O containing 0.1% formic acid (A) and acetonitrile containing 0.1% formic acid (B) were used as eluents applying the following gradient: 0 min: 5% B, 1 min: 5% B, 20 min: 20% B, 40 min: 60% B, 45 min: 95% B, 45.1 min: 5% B and 55 min: 5% B. The DAD detector was set to 210, 254, 280, 320, 330, 350, and 400 nm, the flow rate, injection volume, and column temperature were adjusted to 0.9 mL min^-1^, 20 μL, and 40 °C, respectively. MS spectra were recorded in positive-ESI mode (capillary voltage 4.5 kV), with a drying gas temperature of 300 °C, the nebulizer gas (nitrogen) set to 25 psi, and a nebulizer flow (nitrogen) of 12.0 L min^−1^. The scanned mass range was set between *m*/*z* 50 and 600 (Figures S8, S16, S23, S30, S38, S39 and S47).

## Figures and Tables

**Table 1:** ^1^H NMR data of compounds **1**–**6**.

Position	1 (400 MHz)	2 (600 MHz)	3 (600 MHz)	4 (600 MHz)	5 (600 MHz)	6 (600 MHz)
	*δH* (*J* in Hz)	*δH* (*J* in Hz)	*δH* (*J* in Hz)	*δH* (*J* in Hz)	*δH* (*J* in Hz)	*δH* (*J* in Hz)
4	2.76, d (17.8) 2.83, d (17.8)	2.77, d (17.4) 2.81, d (17.4)	2.76, d (17.4) 2.81, d (17.4)	2.80, d (18.0) 2.83, d (18.0)	2.90, s	2.95, s
6	2.68, d (17.2) 2.97, d (17.2)	2.75, d (17.4) 2.91, d (17.4)	2.67, d (17.4) 2.95, d (17.4)	2.77, d (17.4) 2.90, d (17.4)	2.81, d (18.0) 2.76, d (18.0)	2.74, d (17.4) 2.88, d (17.4)
7	3.57, s	3.57, s	3.56, s	3.56, s	3.58, s	3.59, s
8	3.65, s	3.70, s	3.63, s	3.69, s	3.61, s	3.63, s
9	4.22, dd (8.0,4.8)	4.23, dd (7.8/4.8)	4.23, dd (8.4/4.8)	4.43, dd (8.4/4.8)	3.44, d (14.4/7.8) 3.51, d (14.4/2.4)	3.76, t (6.0)
10					4.02, m	2.78, t (6.0)
11	2.18, m 2.26, m	2.18, m 2.28, m	2.12, m 2.24, m	2.20, m 2.34, m	1.22, d (6.0)	
12	2.45, td (7.62.0)	2.45, td (7.4/2.4)	2.41, m	2.57, td (7.2/1.8)		
1′		4.07, d (4.8)		4.27, d (4.8)	4.43, dd (7.8/5.4)	4.35, m
3′		4.31, m		4.39, m	2.19, m 2.34, m	4.44, m
4′		1.25, d (6.0)		1.25, d (6.6)	2.57, (t, 7.2)	1.25, d (6.6)

**Table 2:** ^13^C NMR data of compounds **1**–**6** and **8**.

Position	1a (400 MHz)	2 (600 MHz)	3 (600 MHz)	4 (600 MHz)	5 (600 MHz)	6 (600 MHz)	8 (600 MHz)
	*δ*_C_, Type	*δ*_C_, Type	*δ*_C_, Type	*δ*_C_, Type	*δ*_C_, Type	*δ*_C_, Type	*δ*_C_, Type
1	163.6, C	162.4, C	163.6, C	163.1, C	161.8, C	161.8, C	174.8, CO
2	127.7, C	128.8, C	127.8, C	129.2, C	128.8, C	128.8, C	81.8, CH
3	163.9, C	162.8, C	164.4, C	163.4, C	164.5, C	164.2, C	28.7, CH_2_
4	36.4, CH_2_	36.2, CH_2_	36.5, CH_2_	36.1, CH_2_	36.1, CH_2_	35.8, CH_2_	25.5, CH_2_
5	74.2, C	74.0, C	74.1, C	74.0, C	74.0, C	74.0, C	30.9, CH_2_
6	38.5, CH_2_	36.0, CH_2_	38.5, CH_2_	36.2, CH_2_	36.0, CH_2_	36.1, CH_2_	41.6, CH_2_
7	70.2, CH_2_	70.3, CH_2_	70.3, CH_2_	70.4, CH_2_	70.4, CH_2_	70.4, CH_2_	180.0, CO
8	61.9, CH_3_	62.4, CH_3_	62.0, CH_3_	62.5, CH_3_	62.2, CH_3_	62.3, CH_3_	40.6, CH_2_
9	61.4, CH	61.5, CH	61.7, CH	60.2, CH	53.1, CH_2_	42.3, CH_2_	22.0, CH_2_
10	179.1, CO	179.3, CO	179.3, CO	178.1, CO	69.5, CH	36.7, CH_2_	15.6, CH_3_
11	30.5, CH_2_	30.6, CH_2_	30.8, CH_2_	29.7, CH_2_	22.3, CH_3_	178.2, CO	54.6, CH_3_
12	34.2, CH_2_	34.3, CH_2_	35.4, CH_2_	33.2, CH_2_			
13	181.1, CO	181.2, CO	182.6 CO	180.3, CO			
1′		67.4, CH		66.3, CH	59.6, CH	65.3, CH	
2′		178.1, CO		176.9, CO	178.0, CO	176.5, CO	
3′		71.1, CH		70.8, CH	29.7, CH_2_	70.6, CH	
4′		22.3, CH_3_		22.2, CH_3_	33.2, CH_2_	22.1, CH_3_	
5′					180.3, CO		

## Supplementary Materials

The following are available online at https://www.mdpi.com/1660-3397/17/6/356/s1, absorption maxima and molecular mass of unidentified MAAs in the methanolic extract of *B. scorpioides*, HRMS spectra, 1D and 2D NMR spectra of all the new compounds **1**–**6** and **8** as well as chromatograms for Marfey’s products of 1–6.
