# Supplementary material for: Bostrychines A–F, Six Novel Mycosporine-Like Amino-Acids and a Novel Betaine from the Red Alga Bostrychia scorpioides
Source: Mar Drugs. 2019 Jun 14;17(6):356. doi: 10.3390/md17060356 (PMC6627687; doi:10.3390/md17060356)
Supplement: Supplementary file 1 [file marinedrugs-17-00356-s001.pdf]

Supplementary Materials

Bostrychines A-F, Six Novel Mycosporine-Like Amino-Acids and a Novel Betaine from the Red Alga *Bostrychia scorpioides*

Maria Orfanoudaki[1], Anja Hartmann[1,*], Helena Miladinovic[1], Hieu Nguyen Ngoc[1], Ulf Karsten[2], and Markus Ganzera[1],

[1] Institute of Pharmacy, Pharmacognosy, University of Innsbruck, Innrain 80-82, Innsbruck 6020, Austria; Maria.Orfanoudaki@uibk.ac.at (M.O.); Anja.Hartmann@uibk.ac.at (A.H.); Helena.Miladinovic@student.uibk.ac.at (H.M.); hieu.nguyen-ngoc@student.uibk.ac.at (H.N.N.); markus.ganzera@uibk.ac.at (M.G.)

[2] Institute of Biological Sciences, Applied Ecology & Phycology, University of Rostock, Albert-Einstein-Str. 3, Rostock 18059, Germany; ulf.karsten@uni-rostock.de

* Correspondence: Anja.Hartmann@uibk.ac.at; Tel.: +43 512 507-58430

Contents

Table S1 Absorption maxima and molecular masses of unidentified compounds in the methanolic extract of *B.scorpioides* 4

Figure S1. ^{1}H NMR spectrum of compound 1 in D_2O 5

Figure S2. COSY spectrum of compound 1 in D_2O 5

Figure S3. HSQC spectrum of compound 1 in D_2O 6

Figure S4. HMBC spectrum of compound 1 in D_2O 6

Figure S5. ^{13}C NMR spectrum of compound 1 in D_2O 7

Figure S6. NOESY spectrum of compound 1 in D_2O 7

Figure S7. High-resolution mass spectrum of compound 1 8

Figure S8. LC-MS of Marfey´s analysis of compound 1 8

Figure S9. LC-MS of the compound 1 9

Figure S10. ^{1}H NMR spectrum of compound 2 in D_2O 10

Figure S11. COSY spectrum of compound 2 in D_2O 10

Figure S12. HSQC spectrum of compound 2 in D_2O 11

Figure S13. HMBC spectrum of compound 2 in D_2O 11

Figure S14. ^{13}C NMR spectrum of compound 2 in D_2O 12

Figure S15. High-resolution mass spectrum of compound 2 12

Figure S16. LC-MS of Marfey´s analysis of compound 2 13

Figure S17. ^{1}H NMR spectrum of compound 3 in D_2O 14

Figure S18. COSY spectrum of compound 3 in D_2O 14

Figure S19. HSQC spectrum of compound 3 in D_2O 15

Figure S20. HMBC spectrum of compound 3 in D_2O 15

Figure S21. ^{13}C NMR spectrum of compound 3 in D_2O 16

Figure S22. High-resolution mass spectrum of compound 3 16

Figure S23. LC-MS of Marfey´s analysis of compound 3 17

Figure S24. ^{1}H NMR spectrum of compound 4 in D_2O 18

Figure S25. COSY spectrum of compound 4 in D_2O 18

Figure S26. HSQC spectrum of compound 4 in D_2O 19

Figure S27. HMBC spectrum of compound 4 in D_2O 20

Figure S28. ^{13}C NMR spectrum of compound 4 in D_2O 20

Figure S29. High-resolution mass spectrum of compound 4 21

Figure S30. LC-MS of Marfey´s analysis of compound 4 22

Figure S31. ^{1}H NMR spectrum of compound 5 in D_2O 23
Figure S32. COSY spectrum of compound 5 in D_2O 23
Figure S33. ^{13}C NMR spectrum of compound 5 in D_2O 24
Figure S34. HSQC spectrum of compound 5 in D_2O 24
Figure S35. HMBC spectrum of compound 5 in D_2O 25
Figure S36. NOESY spectrum of compound 5 in D_2O 25
Figure S37. High-resolution mass spectrum of compound 5 26
Figure S38. LC-MS of Marfey´s analysis of compound 5 (a) 27
Figure S39. LC-MS of Marfey´s analysis of compound 5 (b) 27
Figure S40. ^{1}H NMR spectrum of compound 6 in D_2O 28
Figure S41. COSY spectrum of compound 6 in D_2O 28
Figure S42. HSQC spectrum of compound 6 in D_2O 29
Figure S43. HMBC spectrum of compound 6 in D_2O 29
Figure S44. ^{13}C NMR spectrum of compound 6 in D_2O 30
Figure S45. NOESY spectrum of compound 6 in D_2O 30
Figure S46. High-resolution mass spectrum of compound 6 31
Figure S47. LC-MS of Marfey´s analysis of compound 6 31
Figure S48. ^{1}H NMR spectrum of compound 8 in D_2O 32
Figure S49. COSY spectrum of compound 8 in D_2O 32
Figure S50. HSQC spectrum of compound 8 in D_2O 33
Figure S51. HMBC spectrum of compound 8 in D_2O 33
Figure S52. ^{13}C NMR spectrum of compound 8 in D_2O 34
Figure S53. High-resolution mass spectrum of compound 8 34

Table S1 Absorption maxima and molecular masses of unidentified compounds in the methanolic extract of *B.scorpioides*

Compound	λmax (nm)	Mass (Da)
**i**	331	359
**ii**	331	360
**iii**	308	316
**iv**	332	373
**v**	360	355
**vi**	357	357

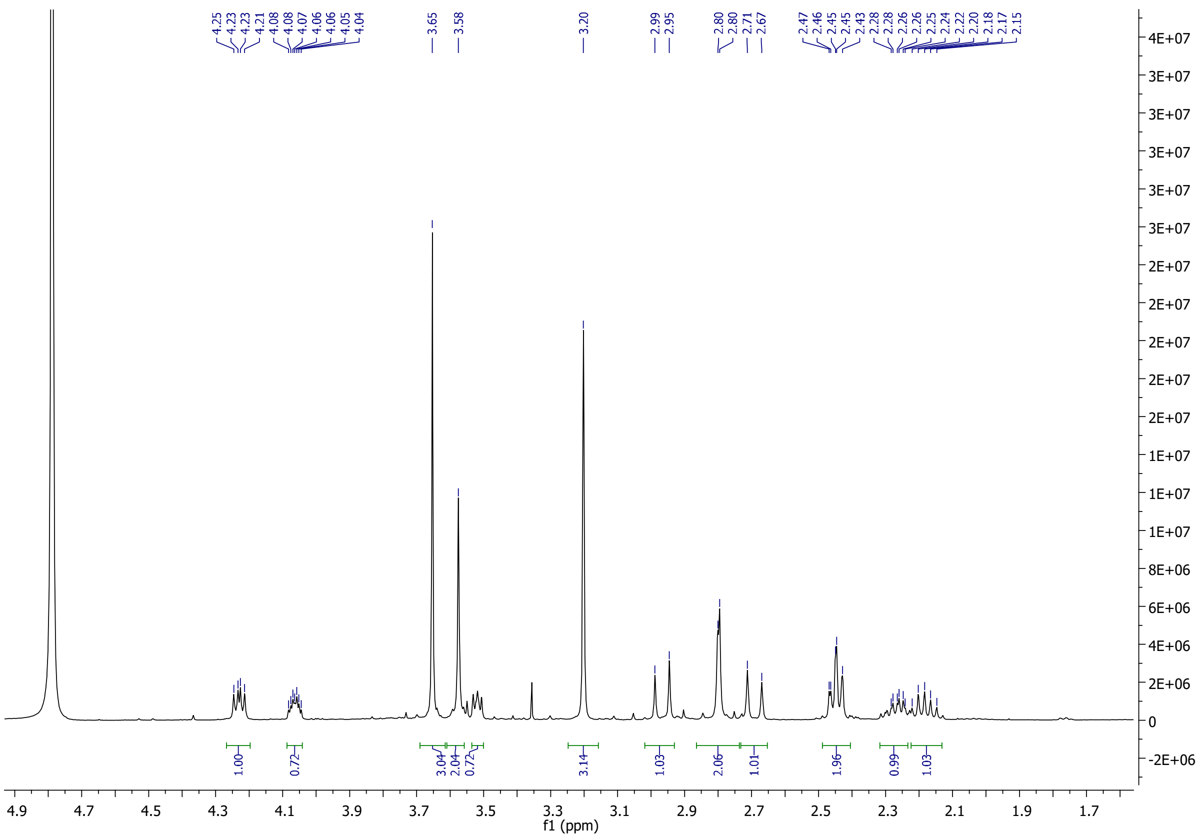

Figure S1. ^{1}H NMR spectrum of compound **1** in D_2O

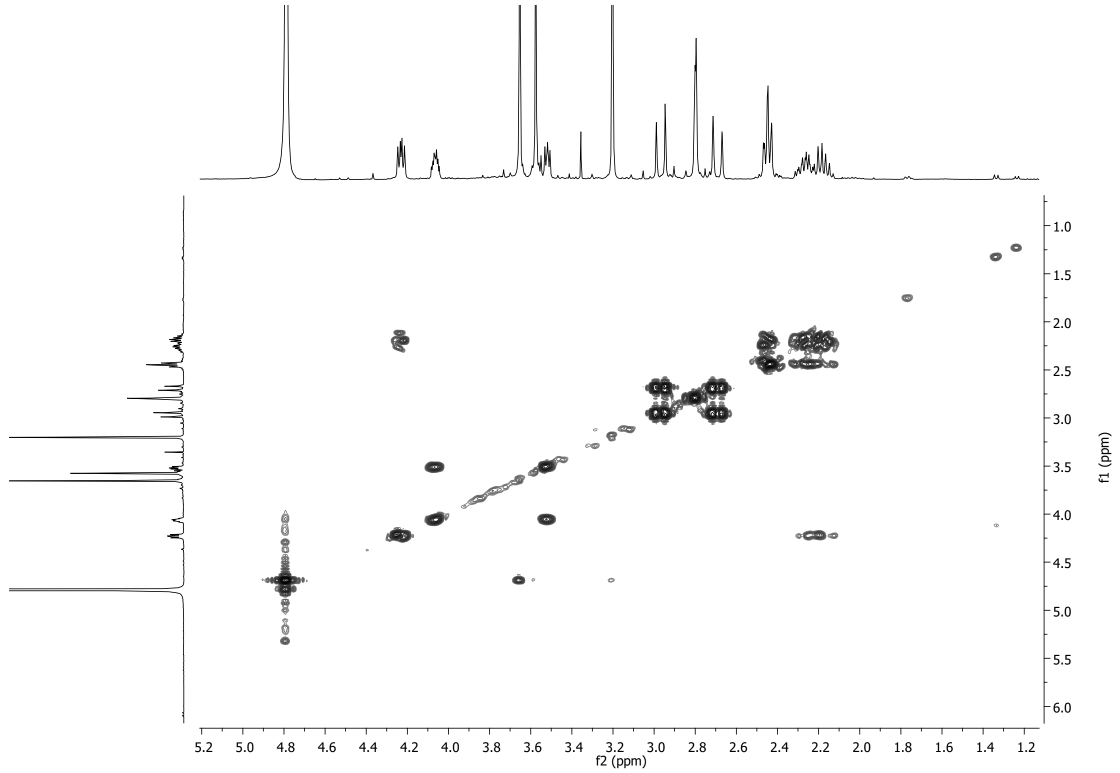

Figure S2. COSY spectrum of compound **1** in D_2O

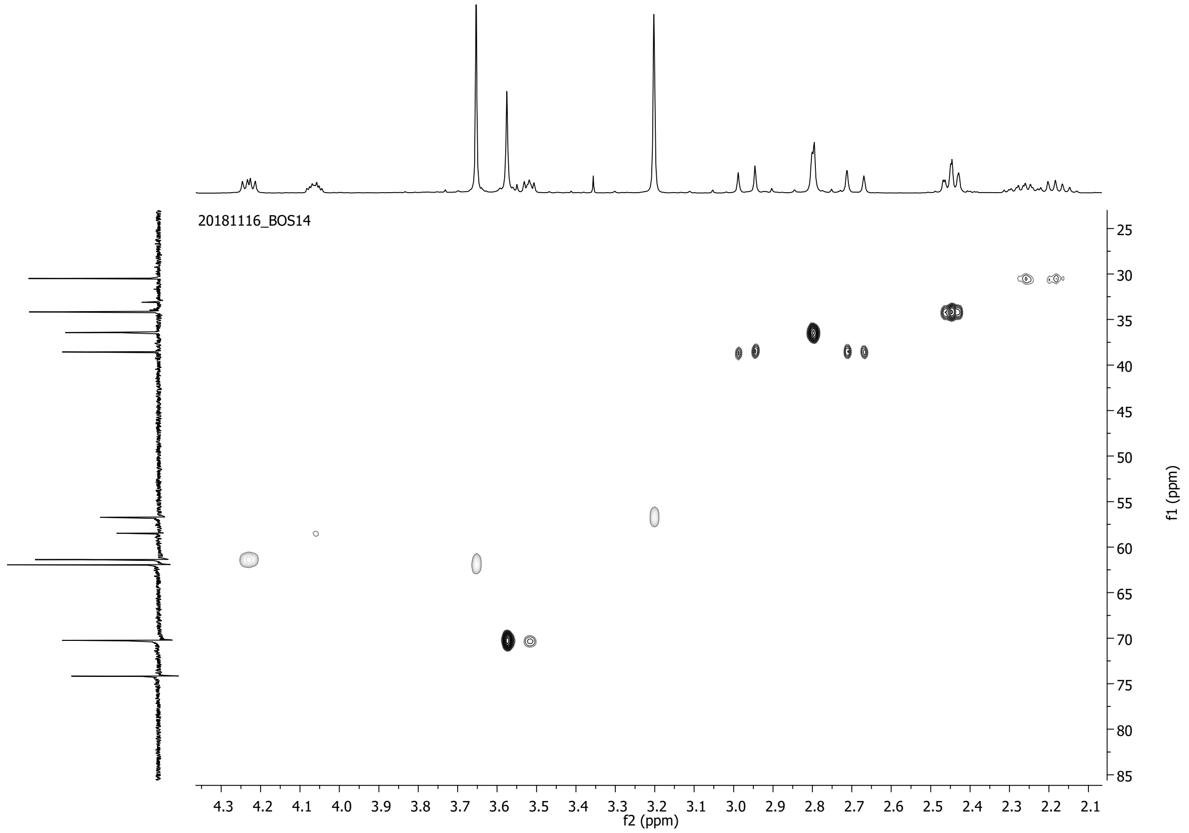

Figure S3. HSQC spectrum of compound **1** in D_2O

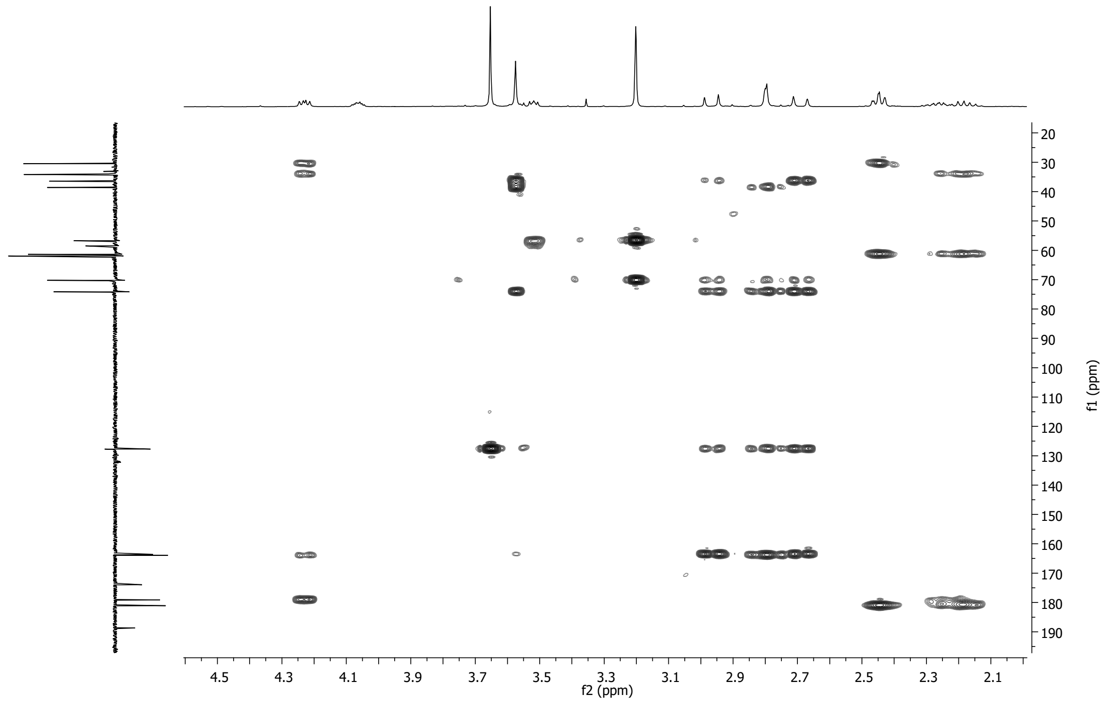

Figure S4. HMBC spectrum of compound **1** in D_2O

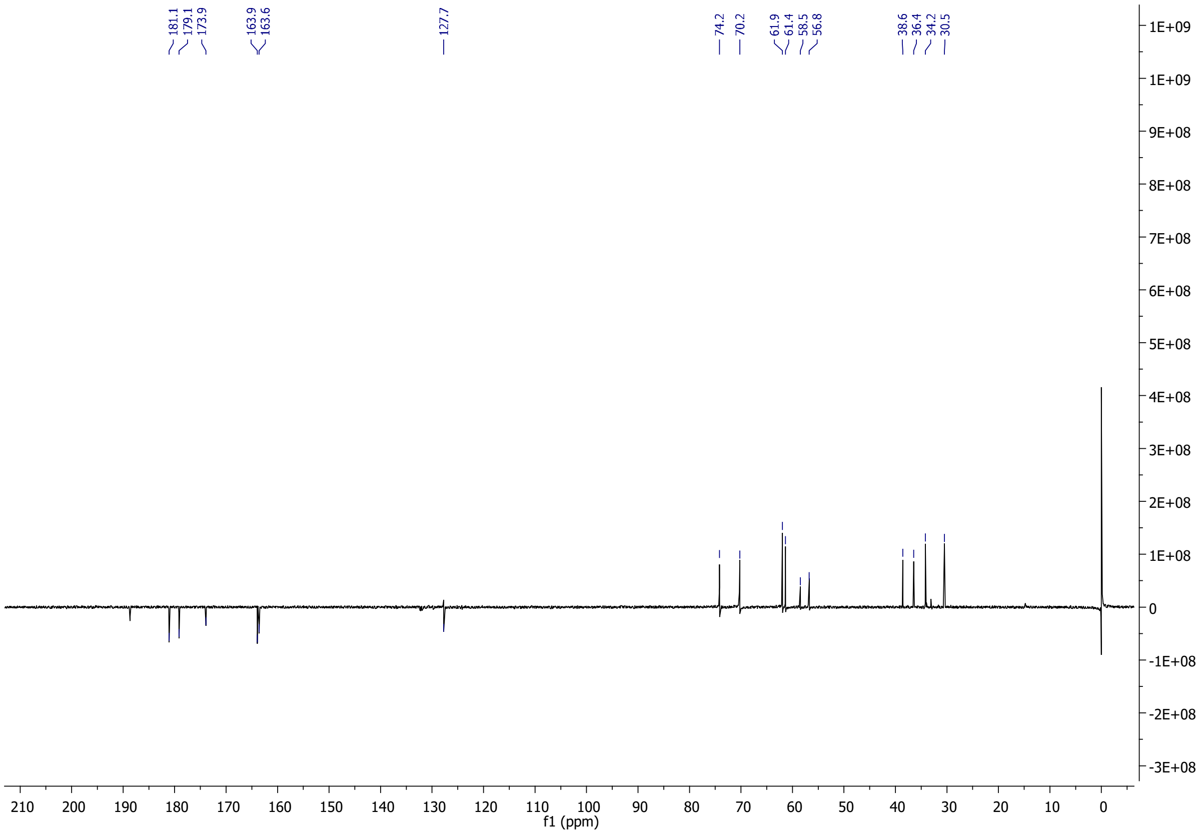

Figure S5. ^{13}C NMR spectrum of compound **1** in D_2O

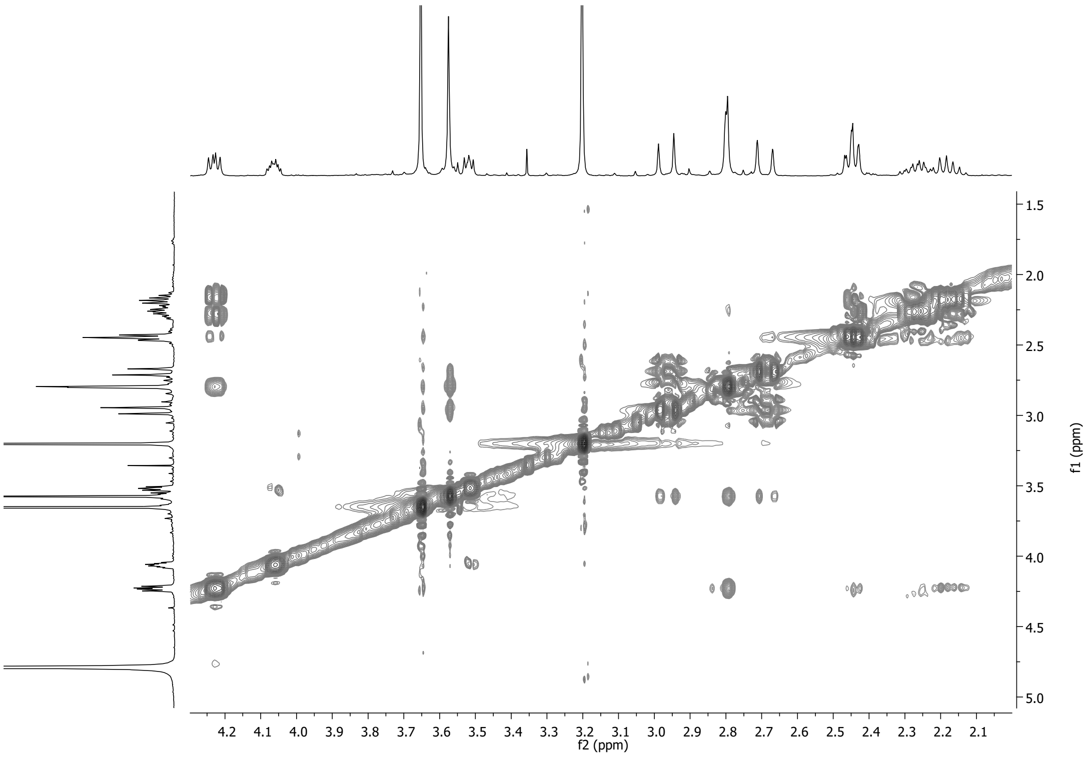

Figure S6. NOESY spectrum of compound **1** in D_2O

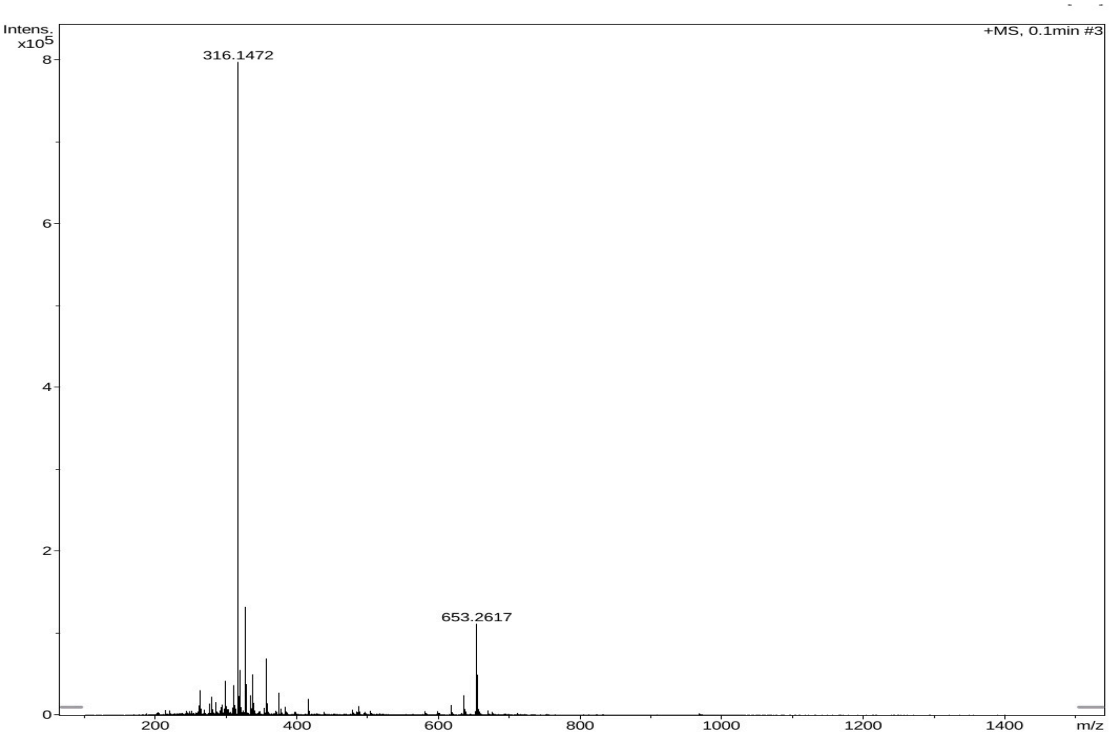

Figure S7. High-resolution mass spectrum of compound **1**

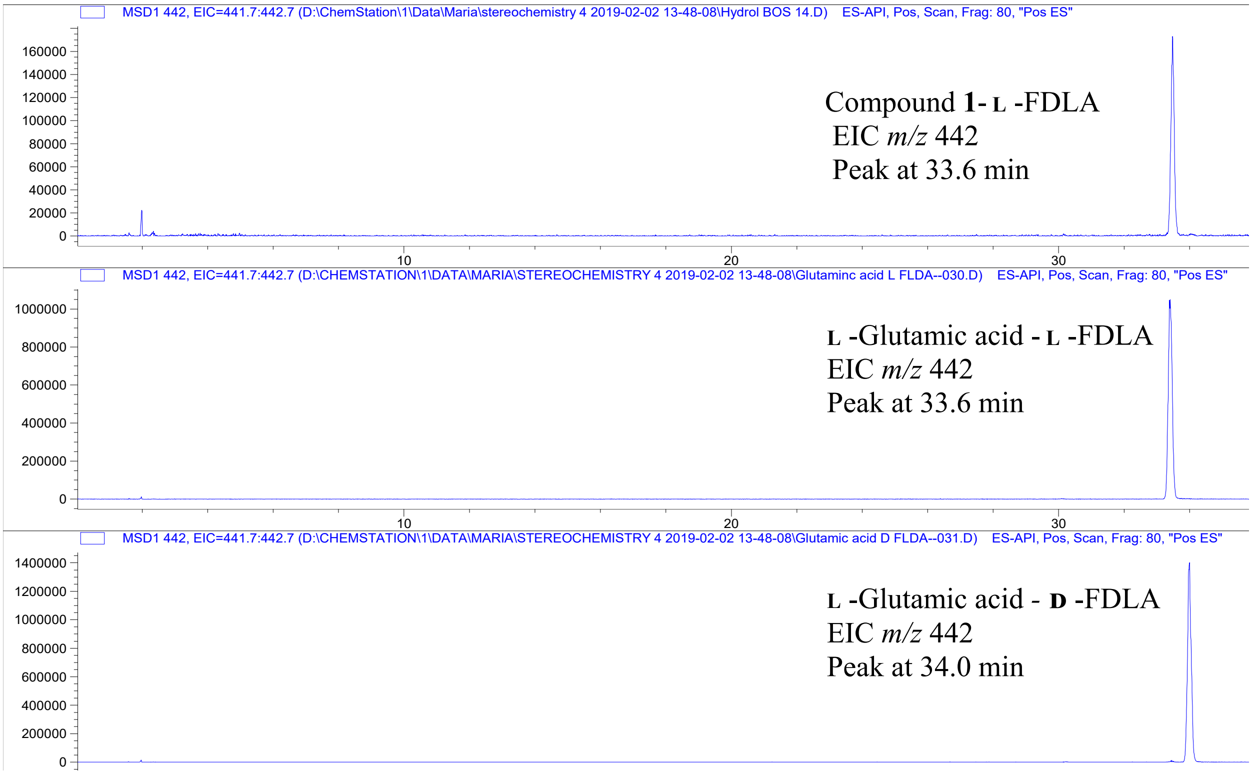

Figure S8. LC-MS of Marfey´s analysis of compound **1**

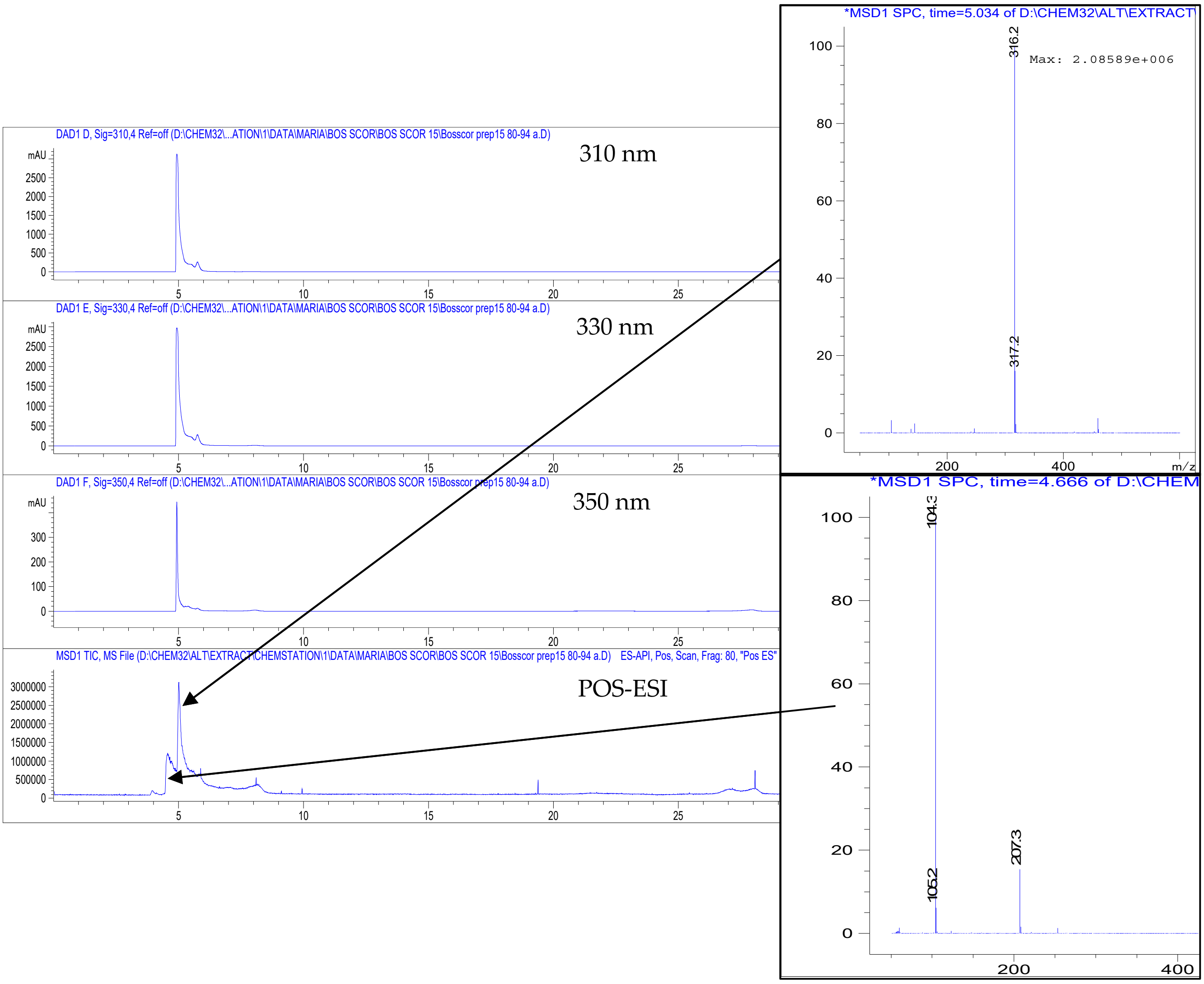

Figure S9. LC-MS of the compound **1**

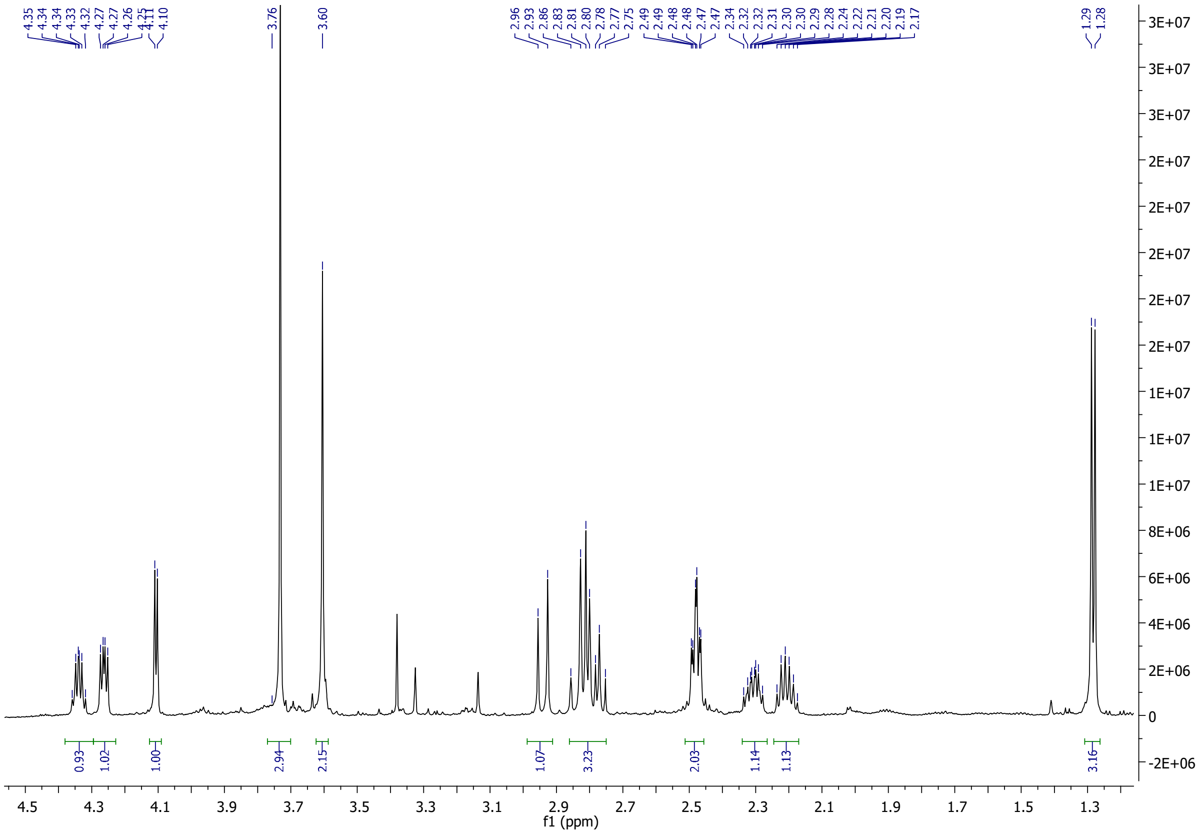

Figure S10. ^{1}H NMR spectrum of compound **2** in D_2O

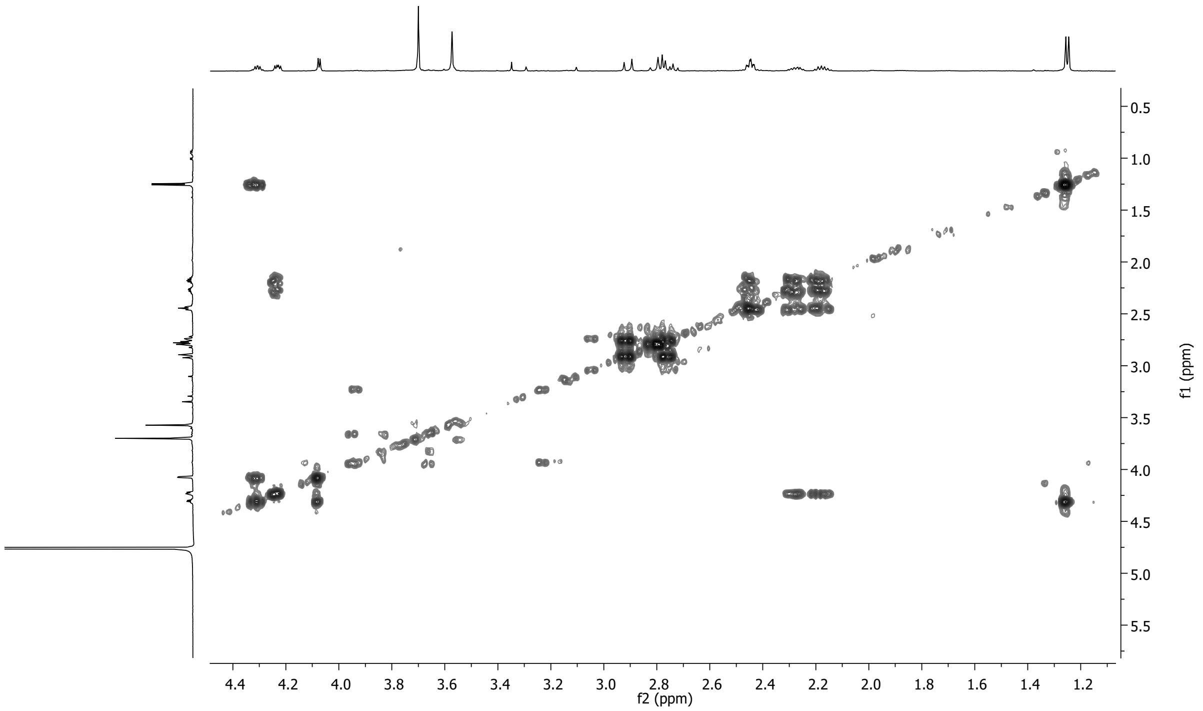

Figure S11. COSY spectrum of compound **2** in D_2O

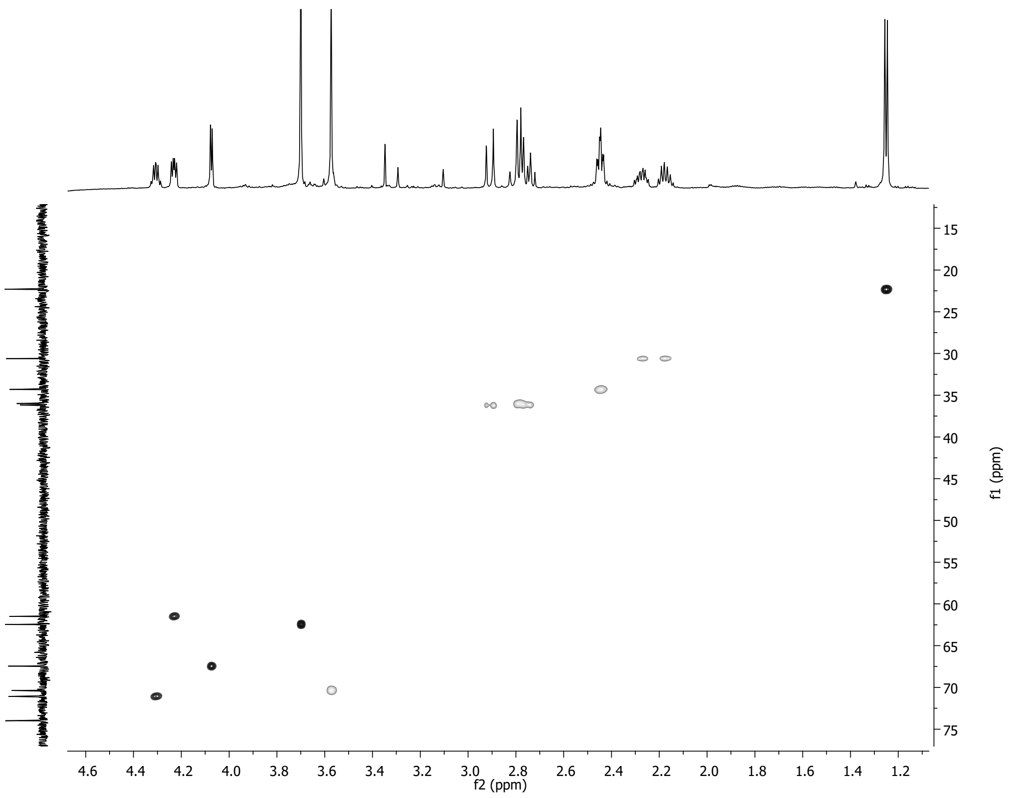

Figure S12. HSQC spectrum of compound **2** in D_2O

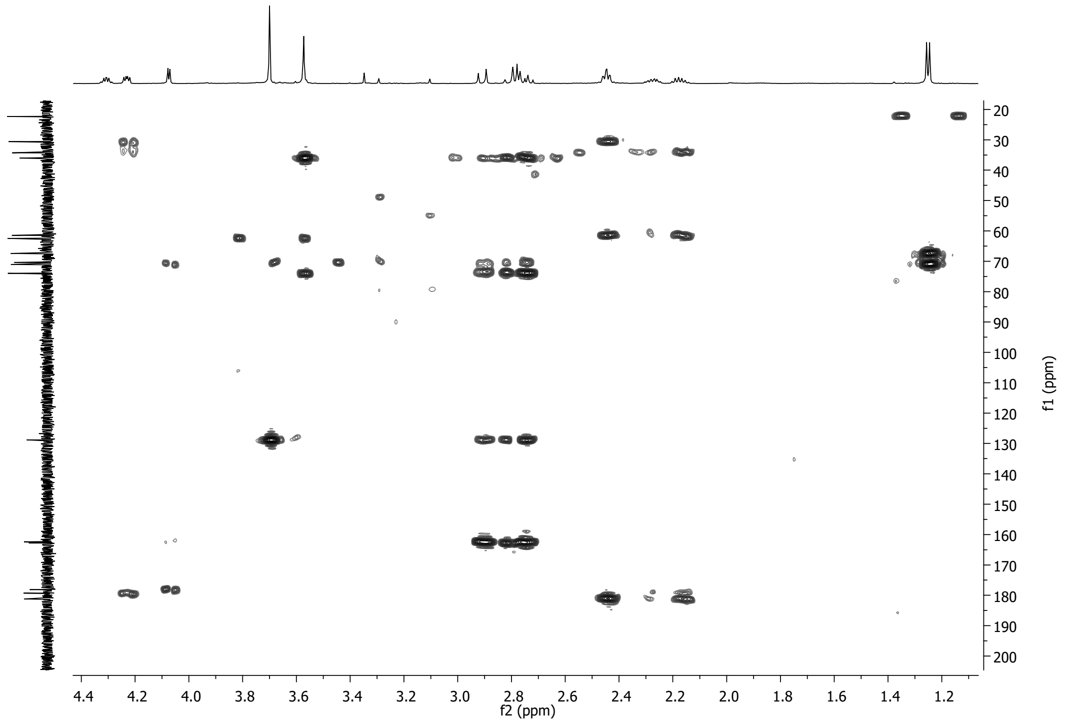

Figure S13. HMBC spectrum of compound **2** in D_2O

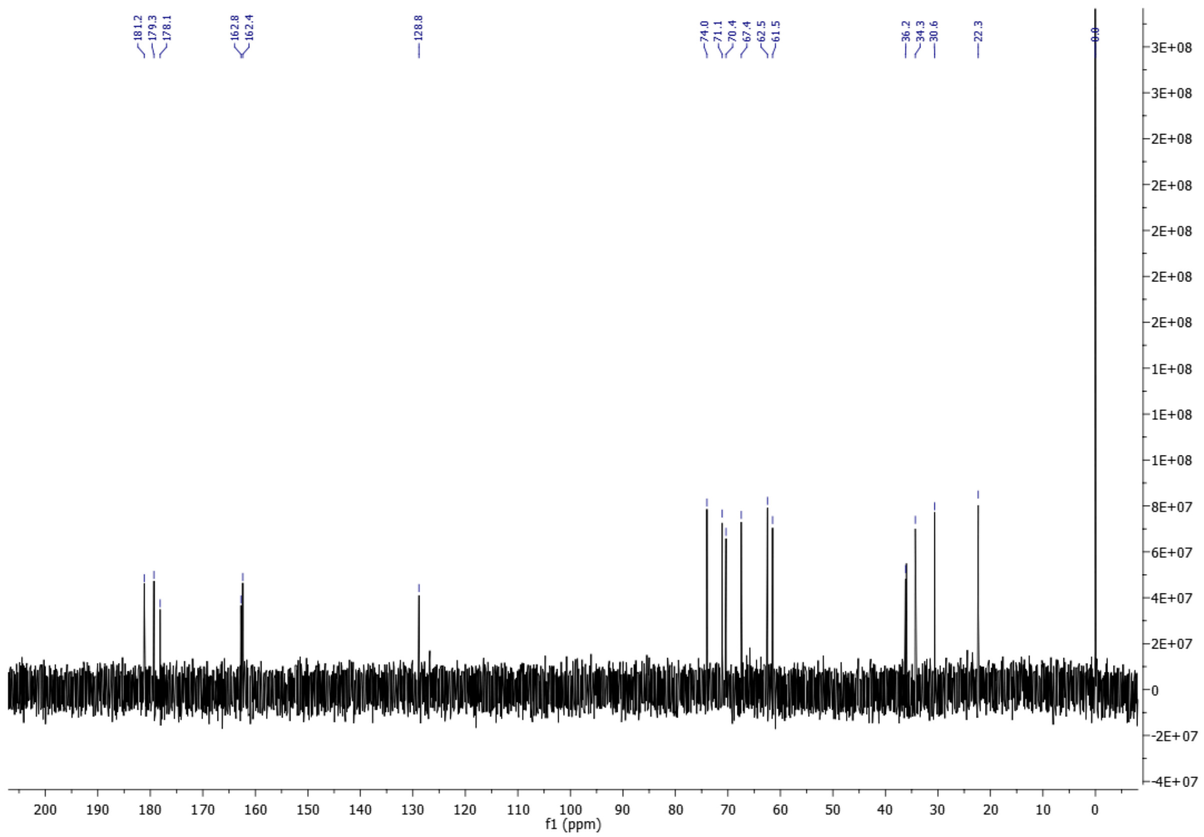

Figure S14. ^{13}C NMR spectrum of compound **2** in D_2O

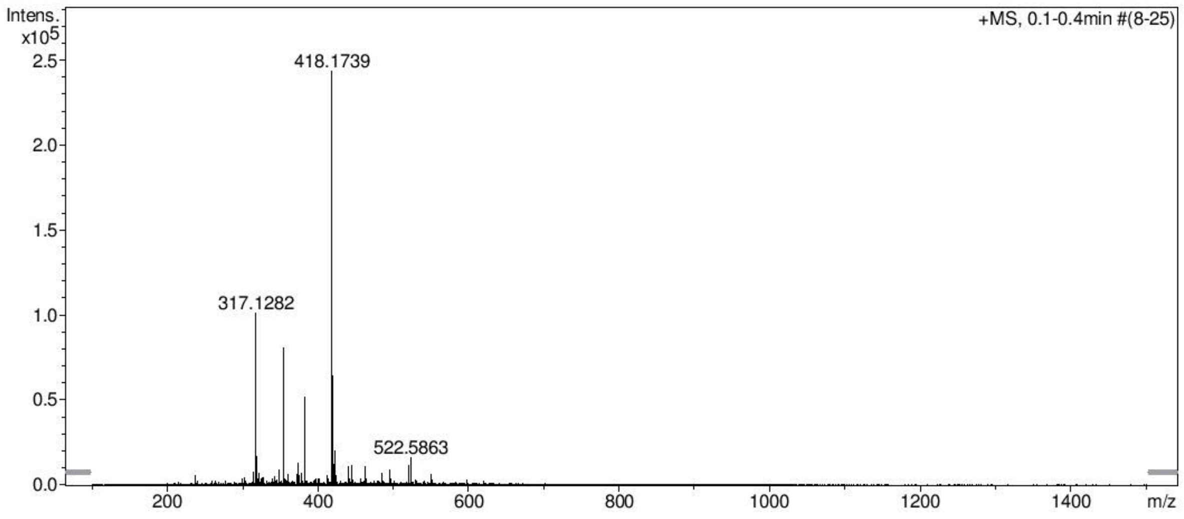

Figure S15. High-resolution mass spectrum of compound **2**

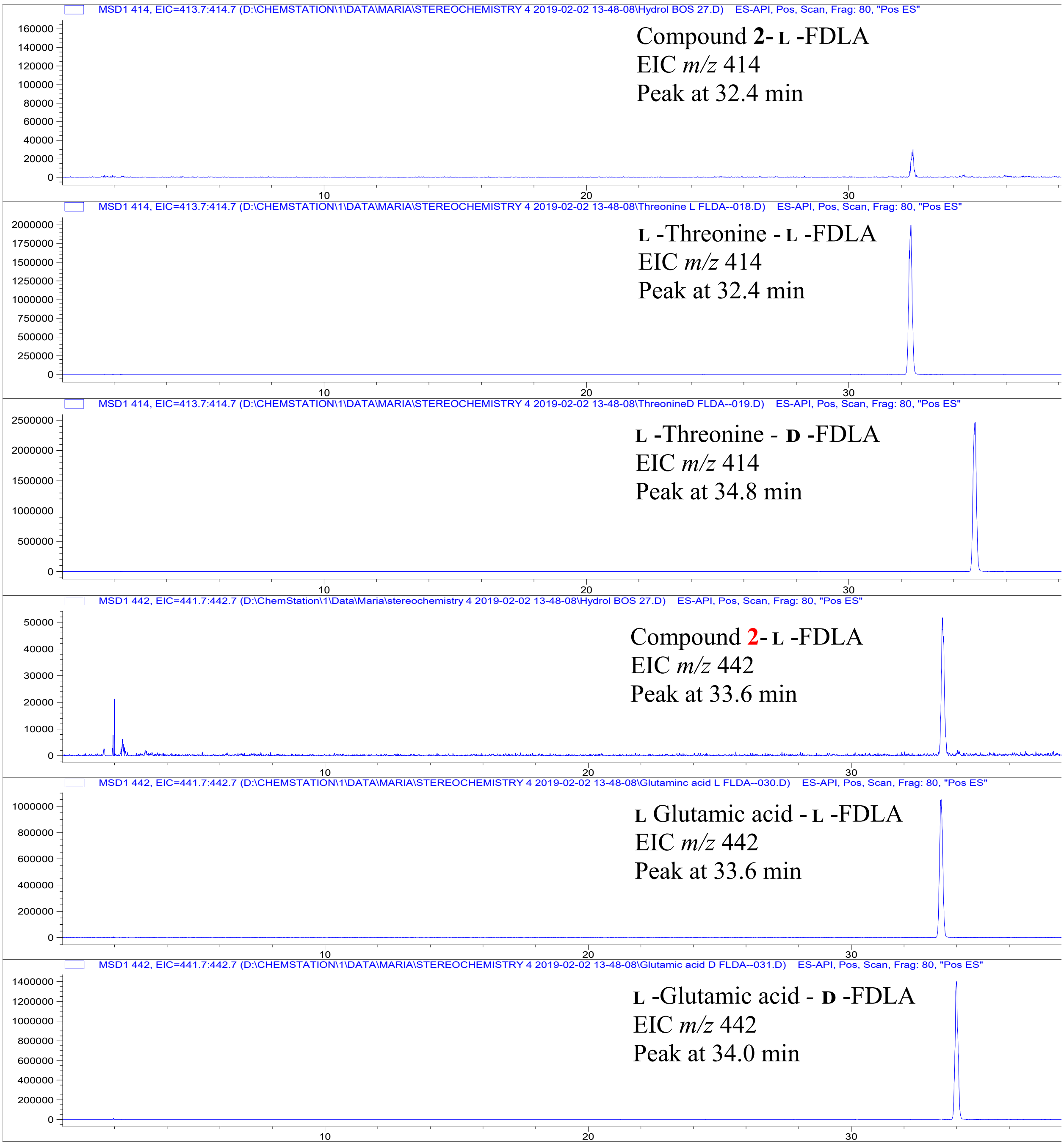

Figure S16. LC-MS of Marfey´s analysis of compound **2**

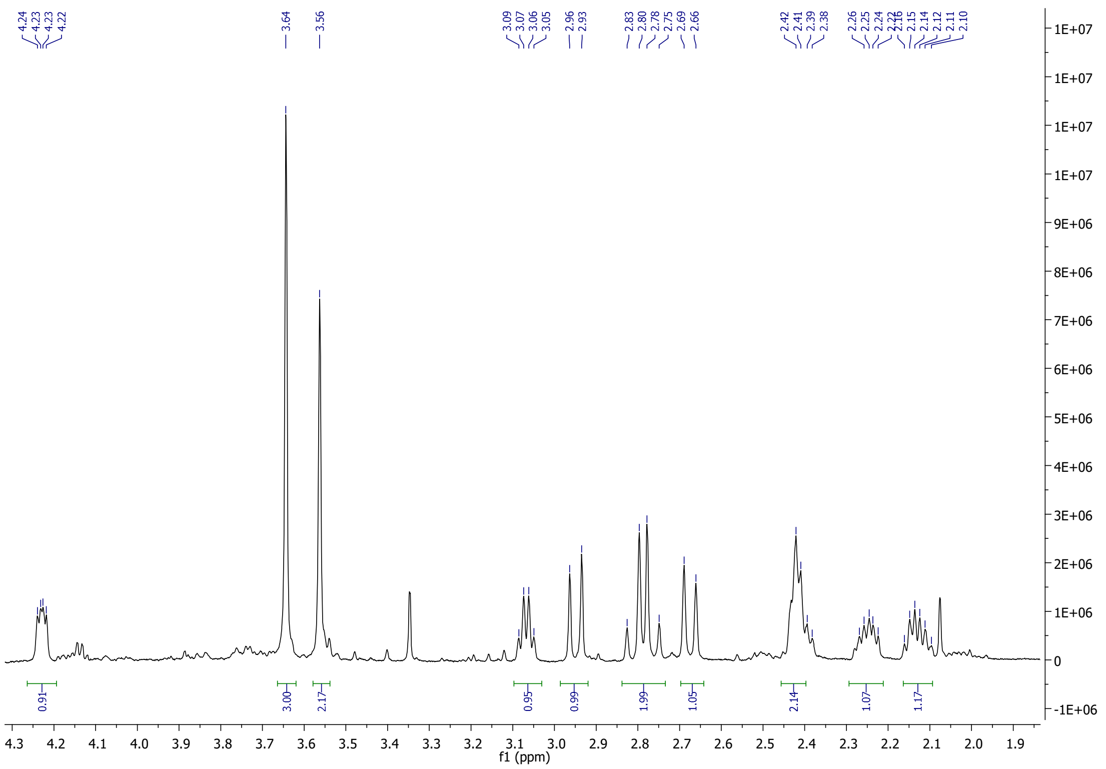

Figure S17. ^{1}H NMR spectrum of compound **3** in D_2O

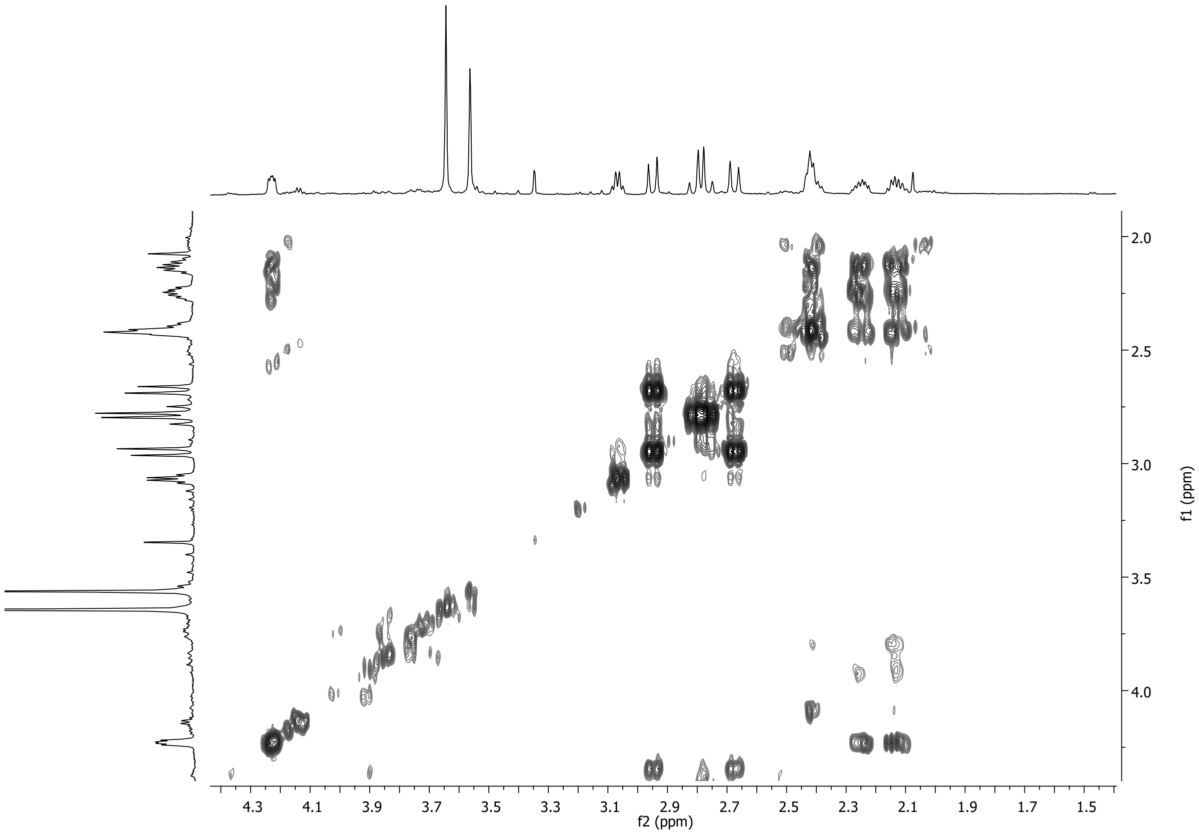

Figure S18. COSY spectrum of compound **3** in D_2O

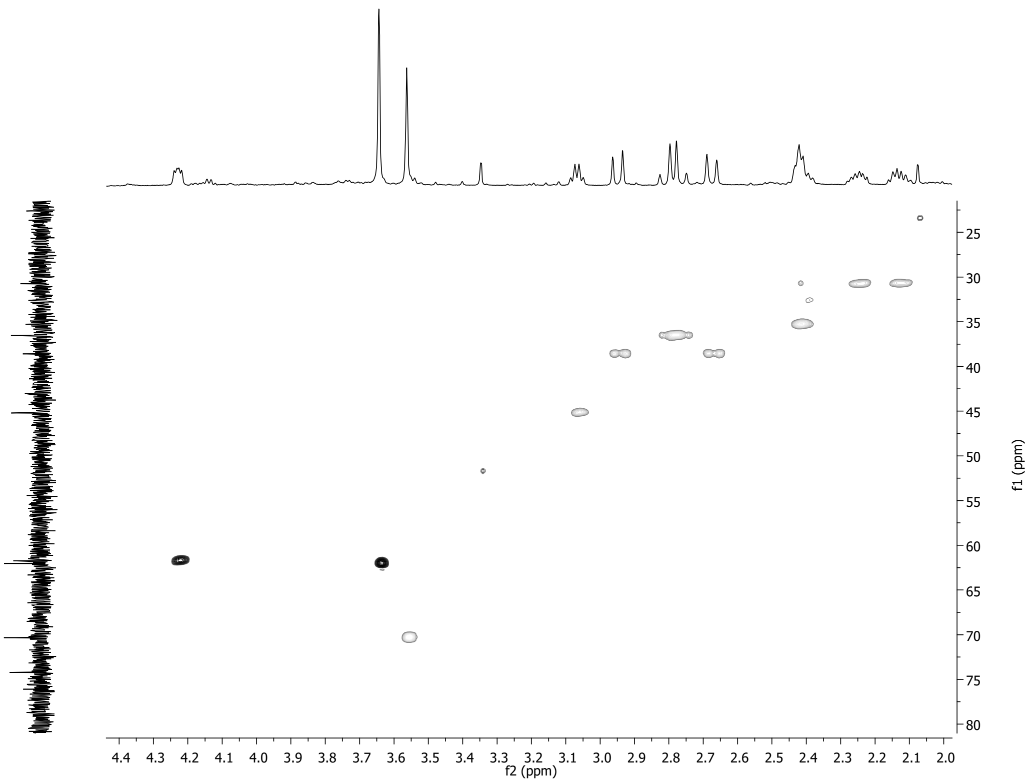

Figure S19. HSQC spectrum of compound **3** in D_2O

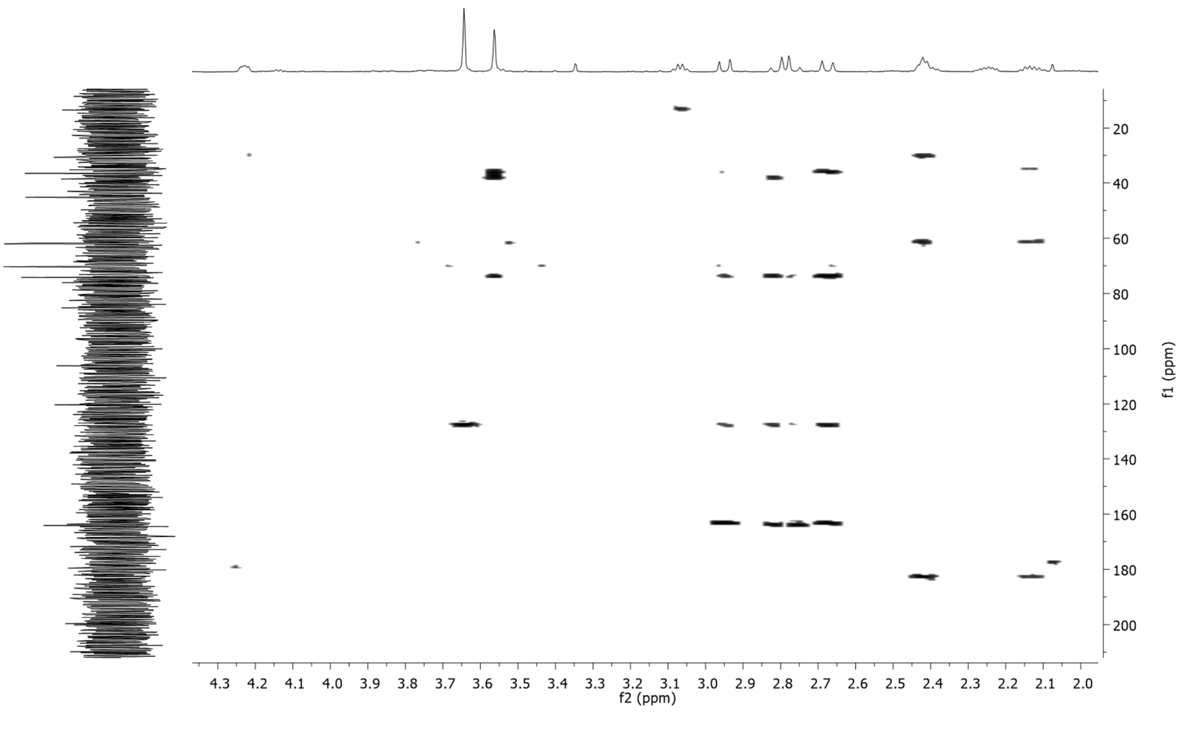

Figure S20. HMBC spectrum of compound **3** in D_2O

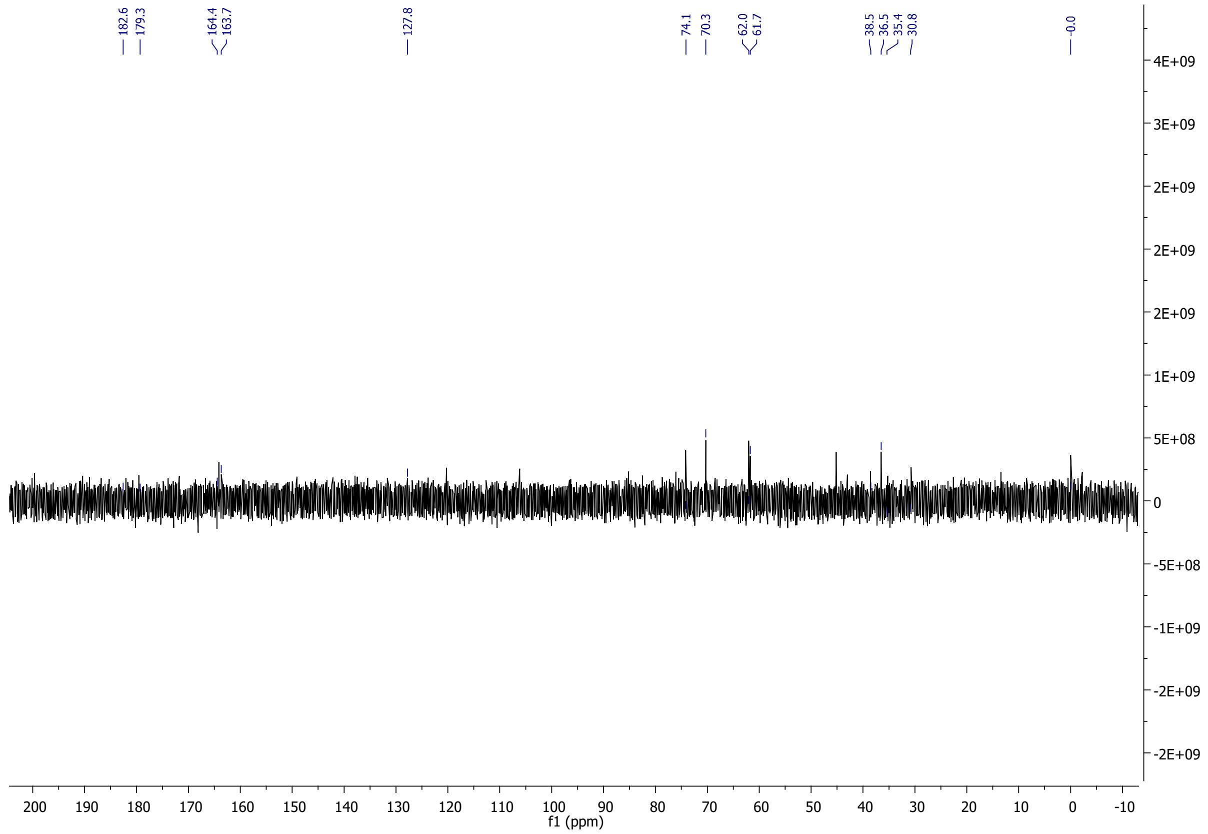

Figure S21. ^{13}C NMR spectrum of compound **3** in D_2O

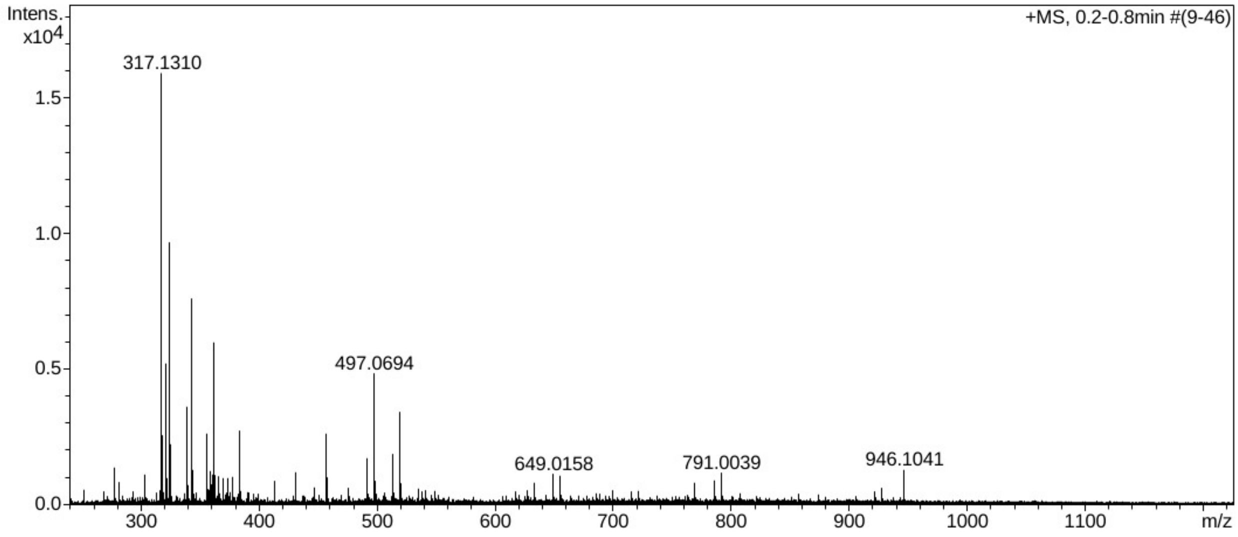

Figure S22. High-resolution mass spectrum of compound **3**

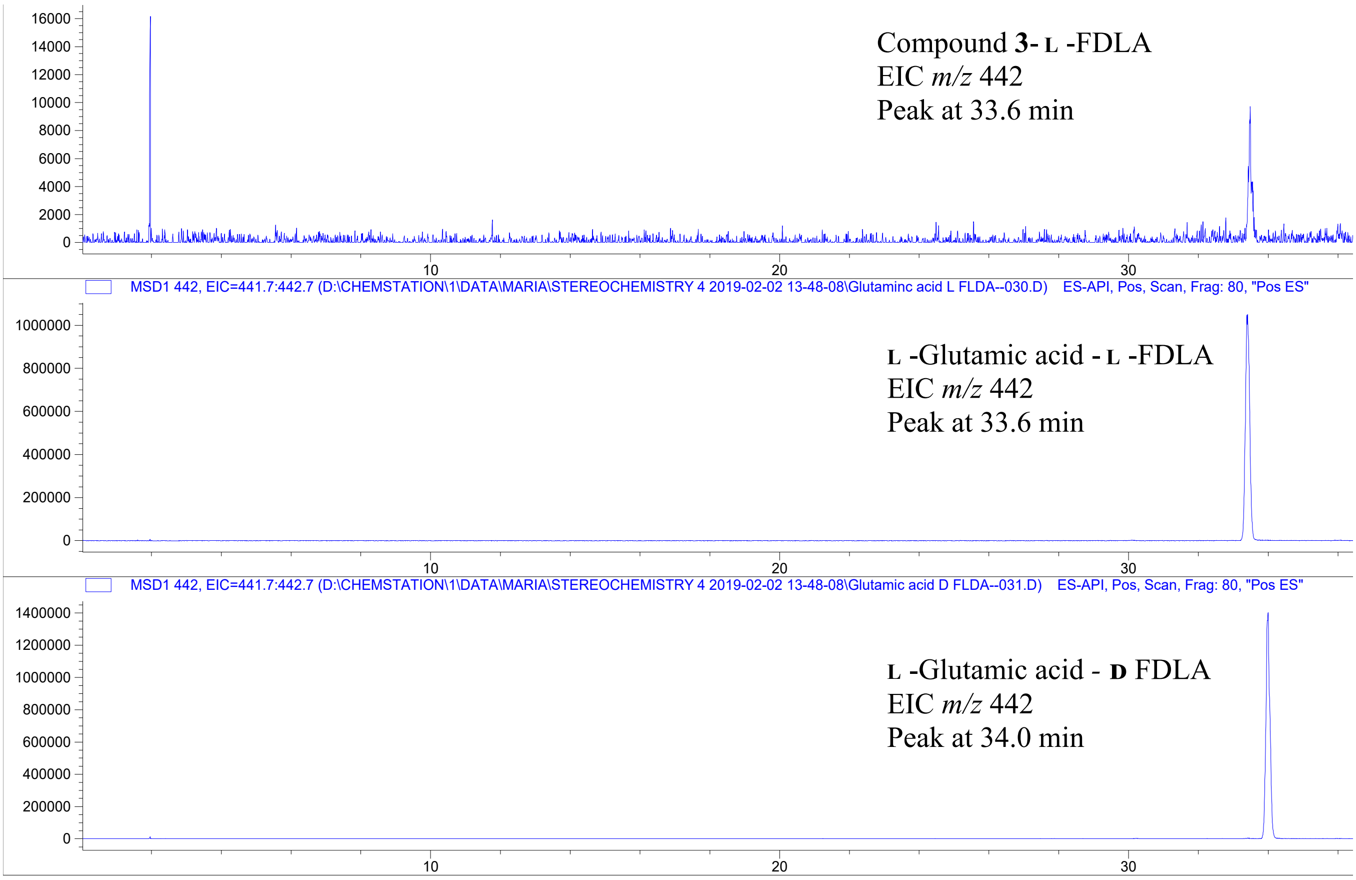

Figure S23. LC-MS of Marfey´s analysis of compound **3**

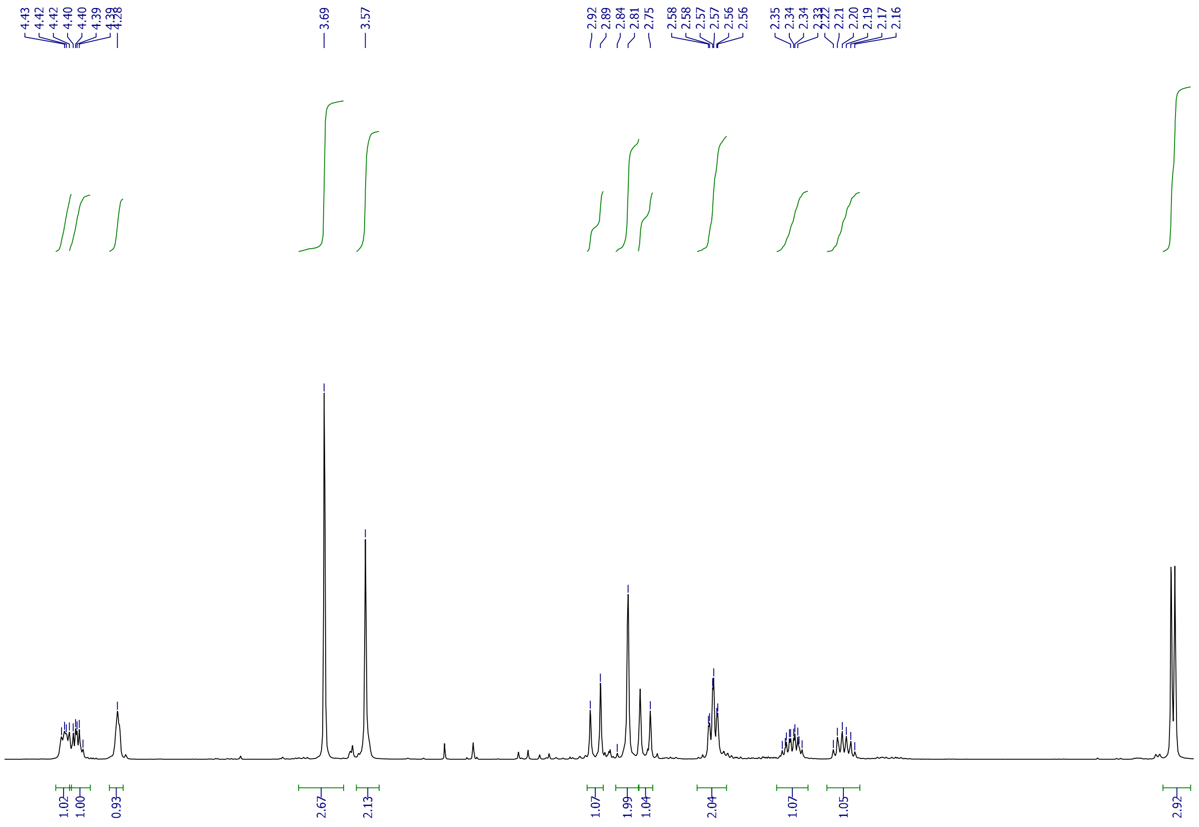

Figure S24. ^{1}H NMR spectrum of compound **4** in D_2O

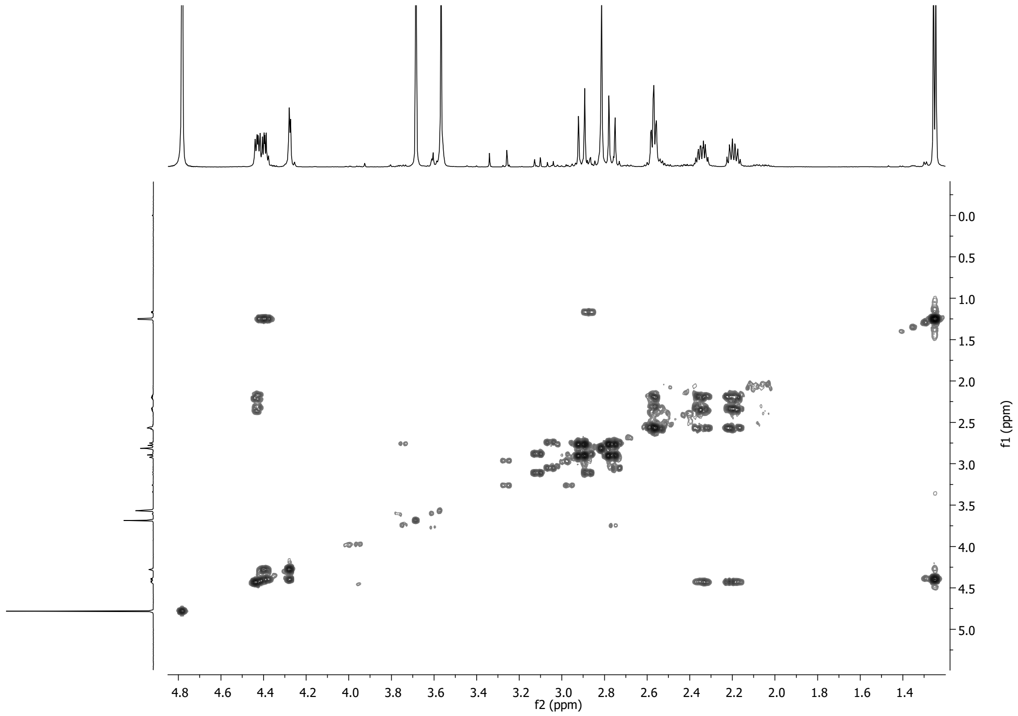

Figure S25. COSY spectrum of compound **4** in D_2O

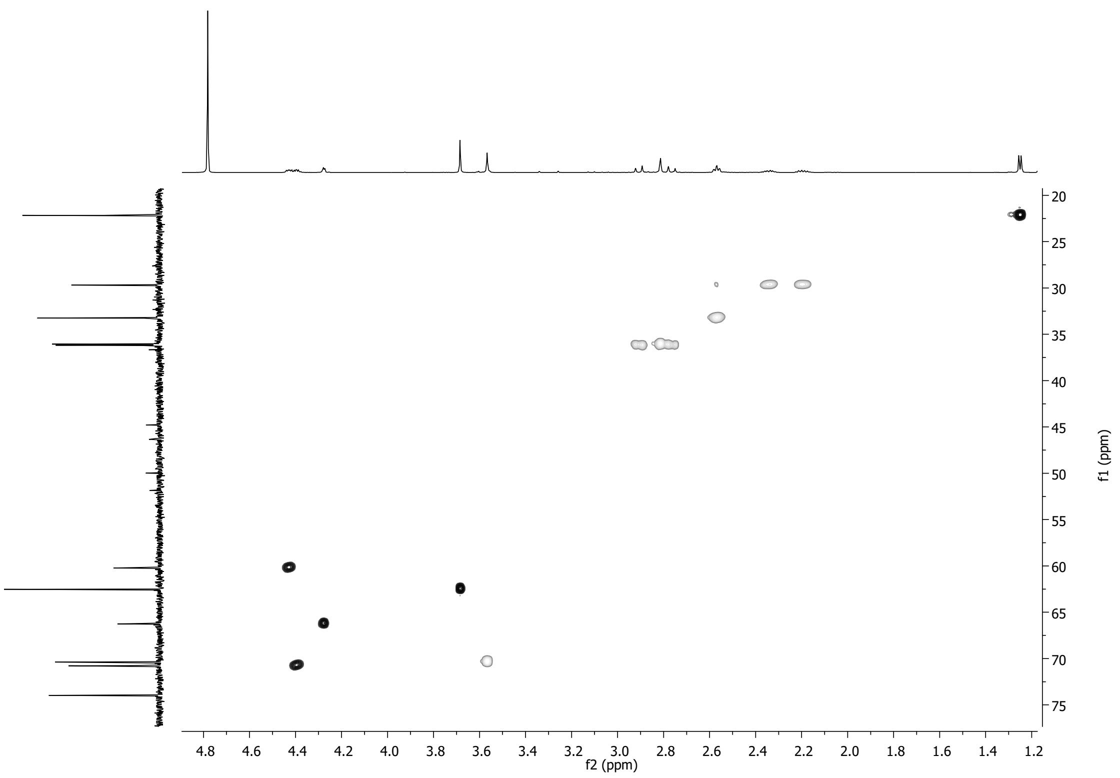

Figure S26. HSQC spectrum of compound **4** in D_2O

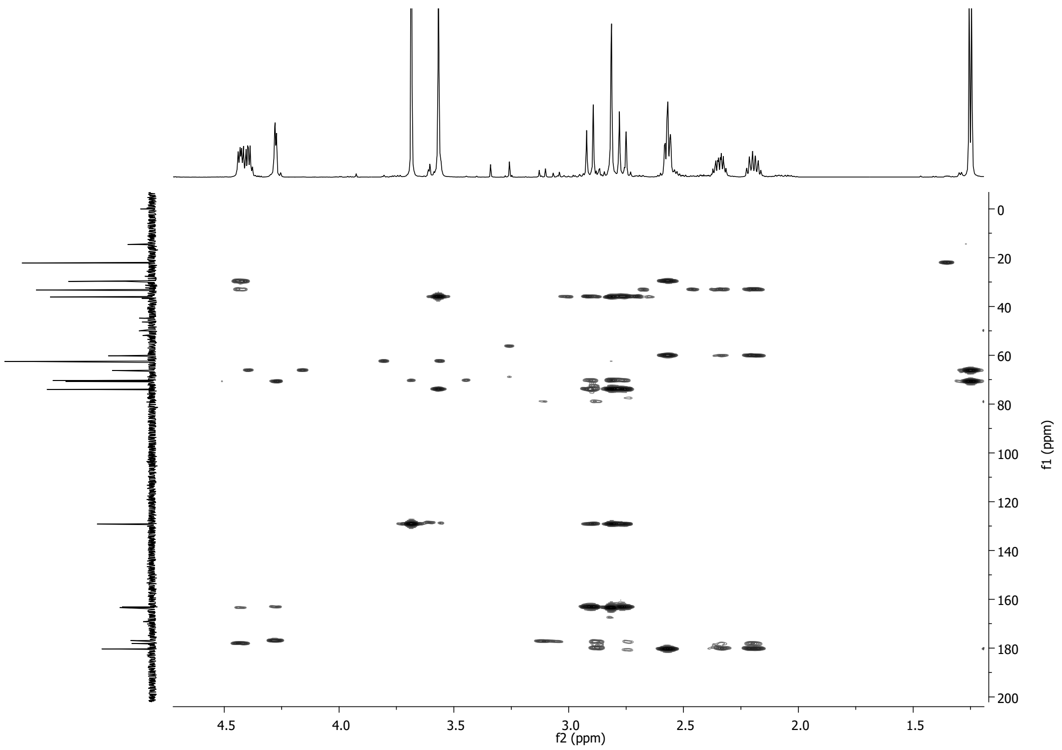

Figure S27. HMBC spectrum of compound **4** in D_2O

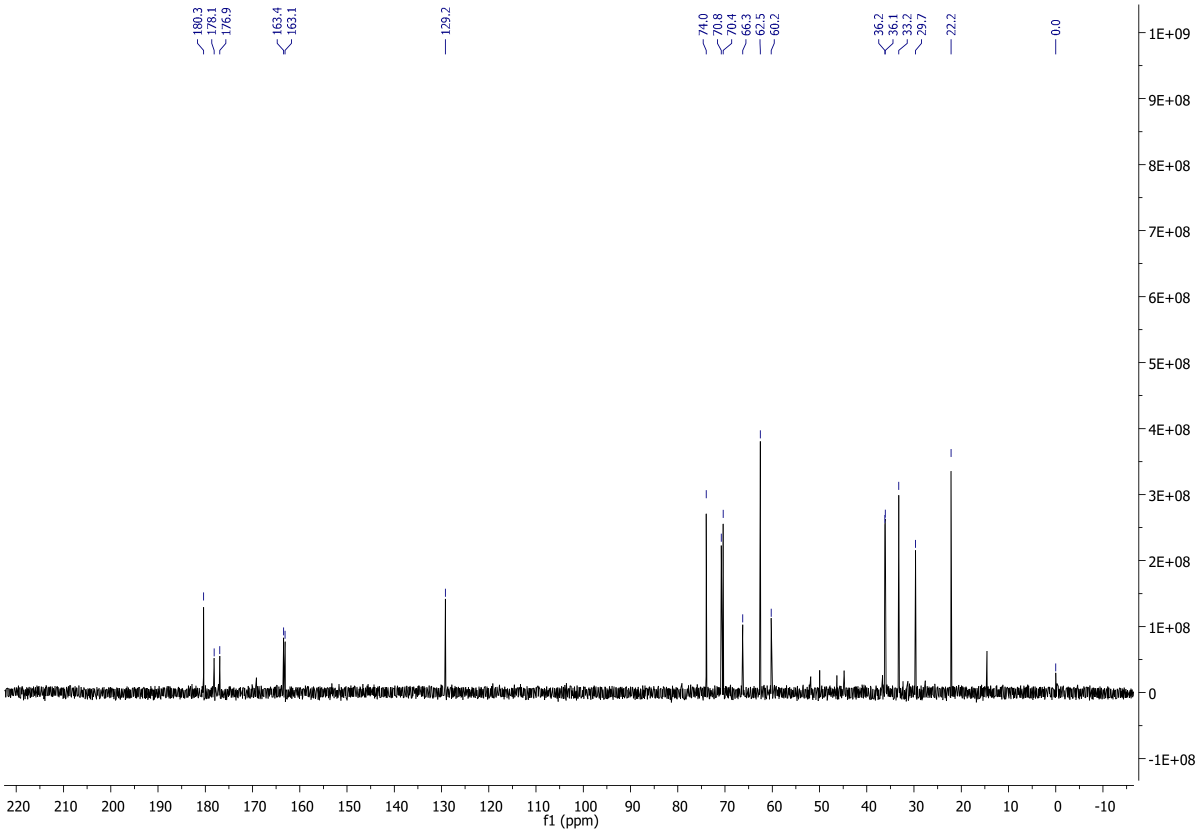

Figure S28. ^{13}C NMR spectrum of compound **4** in D_2O

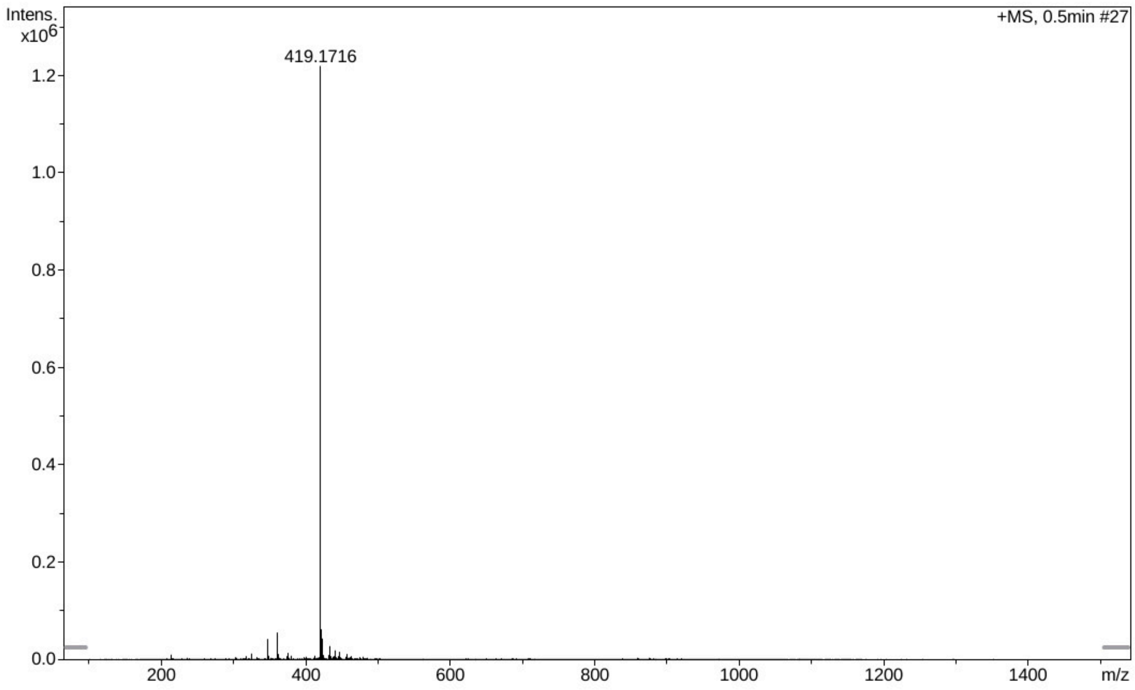

Figure S29. High-resolution mass spectrum of compound **4**

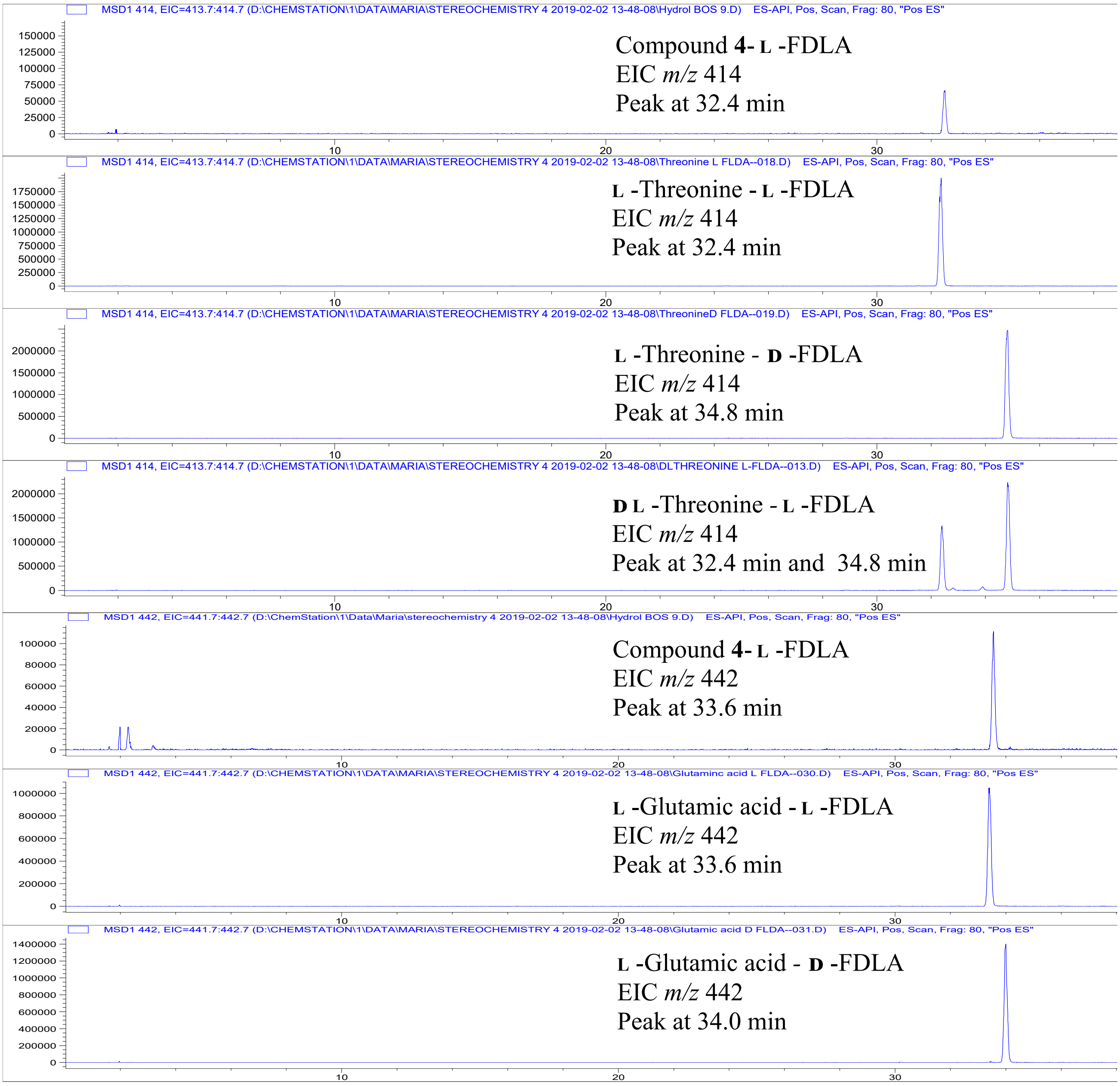

Figure S30. LC-MS of Marfey´s analysis of compound **4**

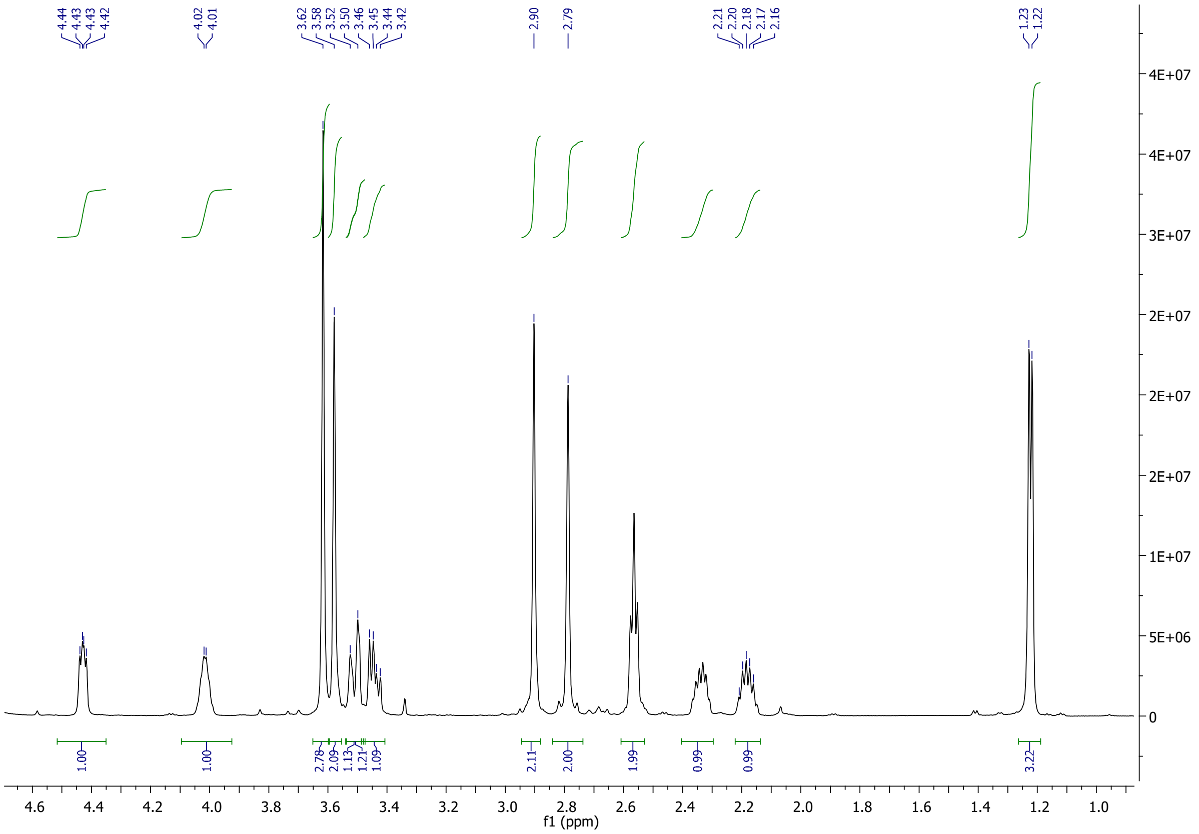

Figure S31. ^{1}H NMR spectrum of compound **5** in D_2O

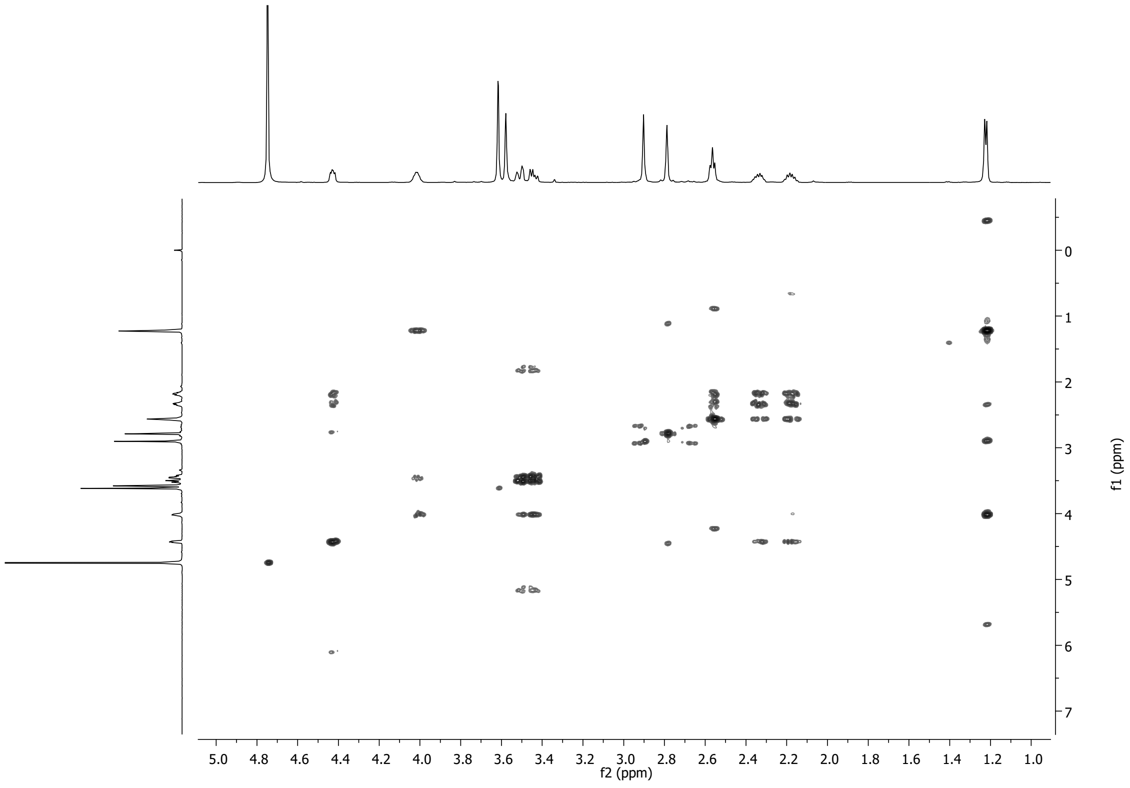

Figure S32. COSY spectrum of compound **5** in D_2O

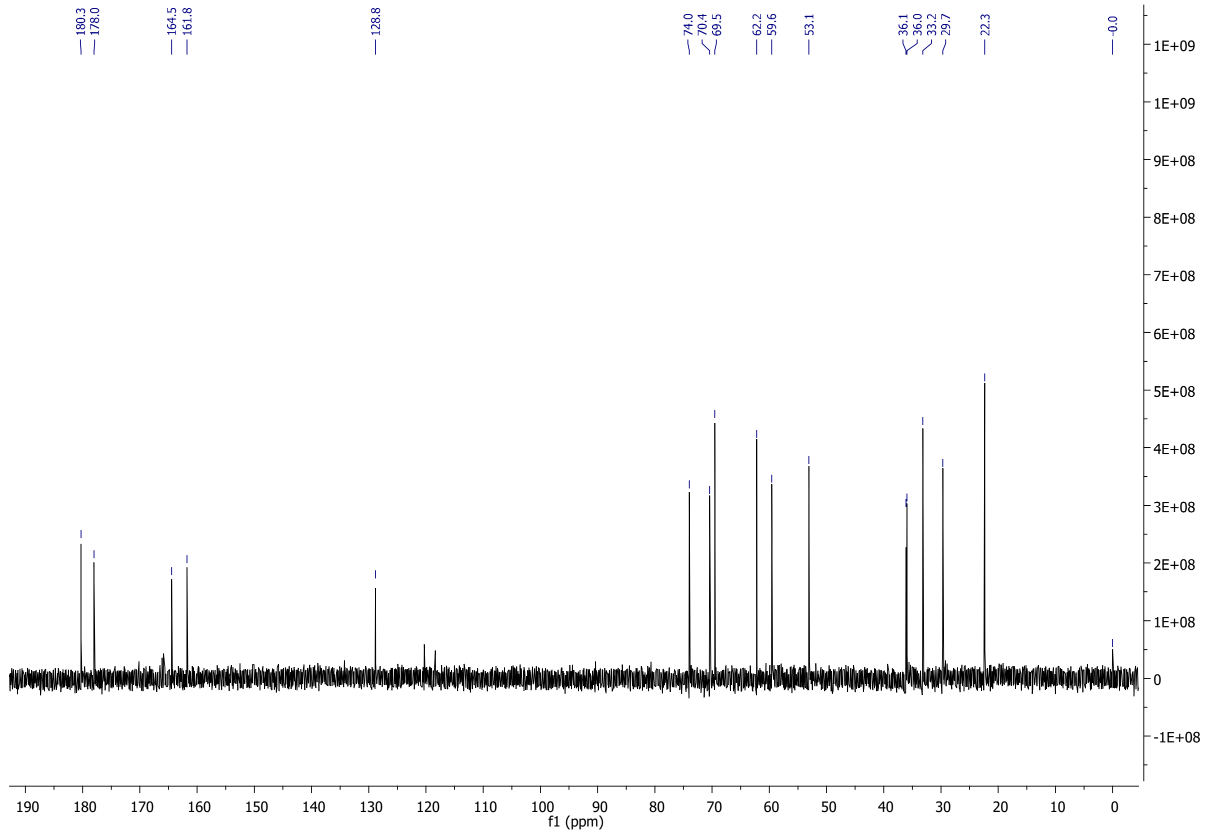

Figure S33. ^{13}C NMR spectrum of compound **5** in D_2O

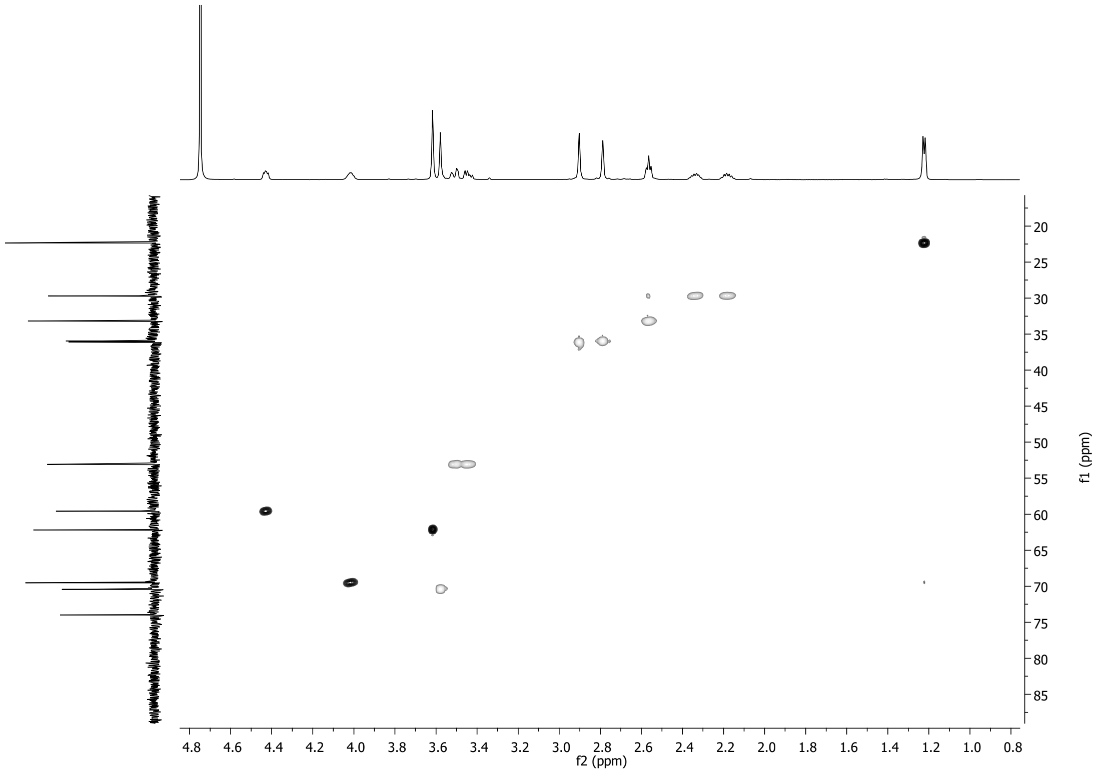

Figure S34. HSQC spectrum of compound **5** in D_2O

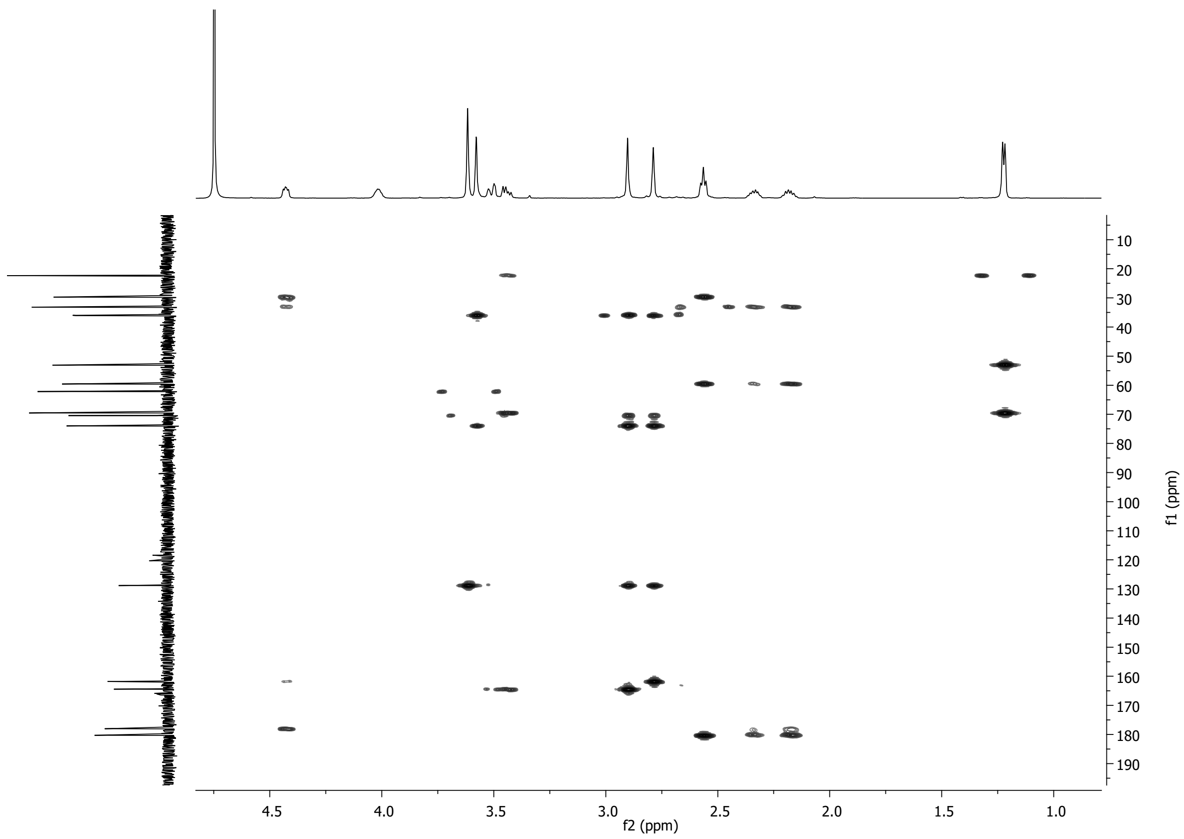

Figure S35. HMBC spectrum of compound **5** in D_2O

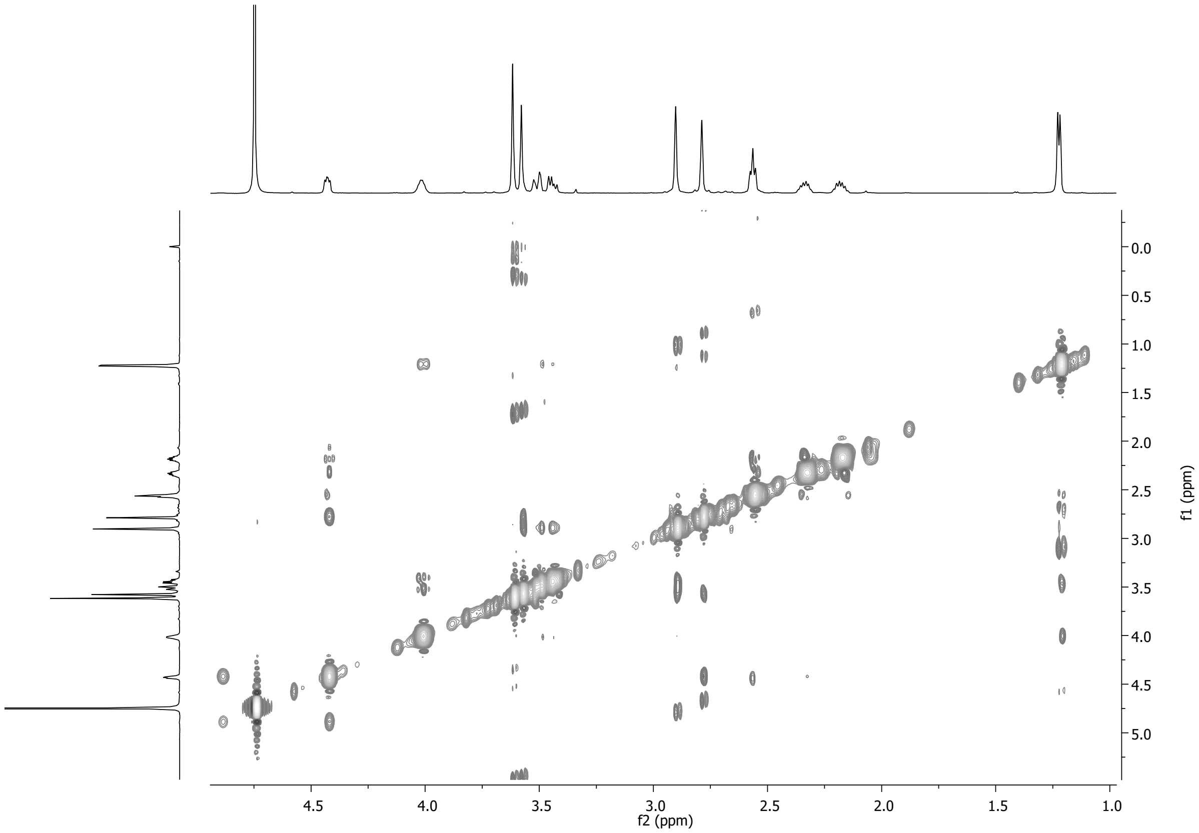

Figure S36. NOESY spectrum of compound **5** in D_2O

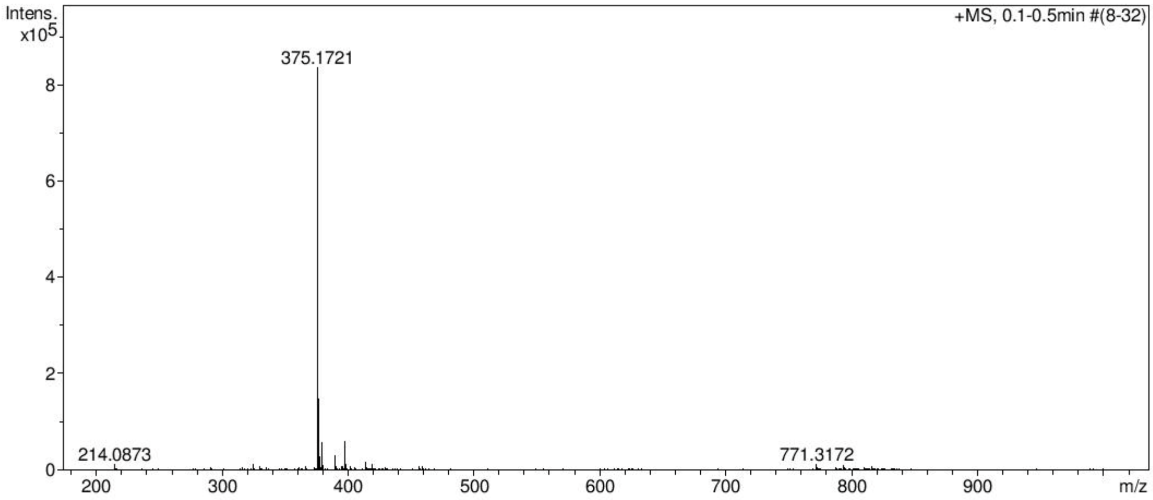

Figure S37. High-resolution mass spectrum of compound **5**

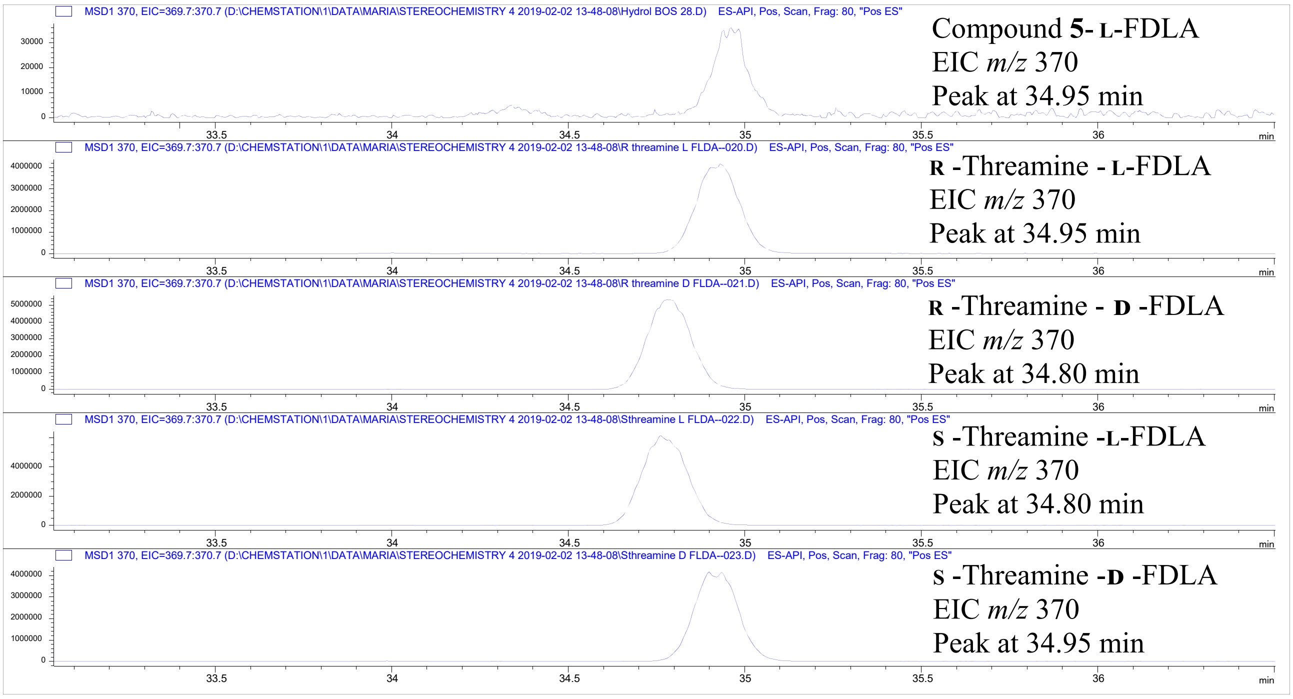

Figure S38. LC-MS of Marfey´s analysis of compound **5** (a)

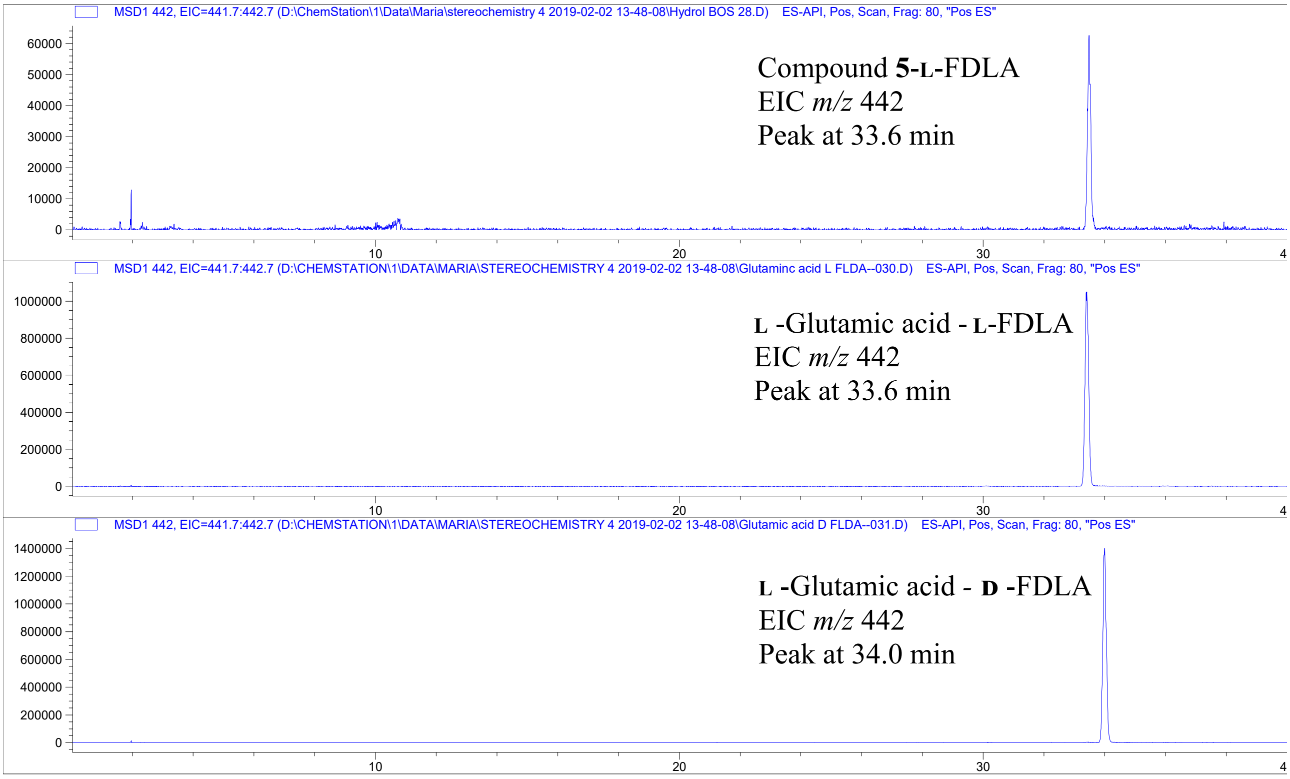

Figure S39. LC-MS of Marfey´s analysis of compound **5** (b)

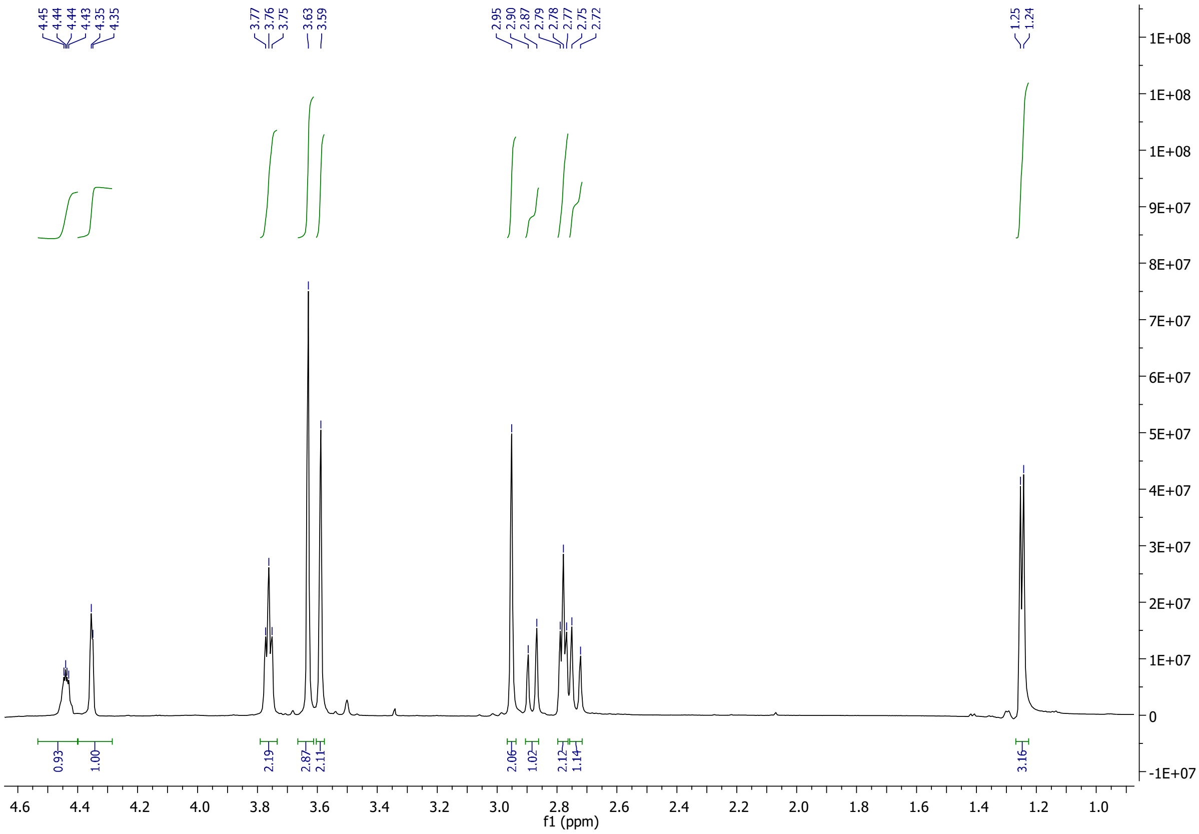

Figure S40. ^{1}H NMR spectrum of compound **6** in D_2O

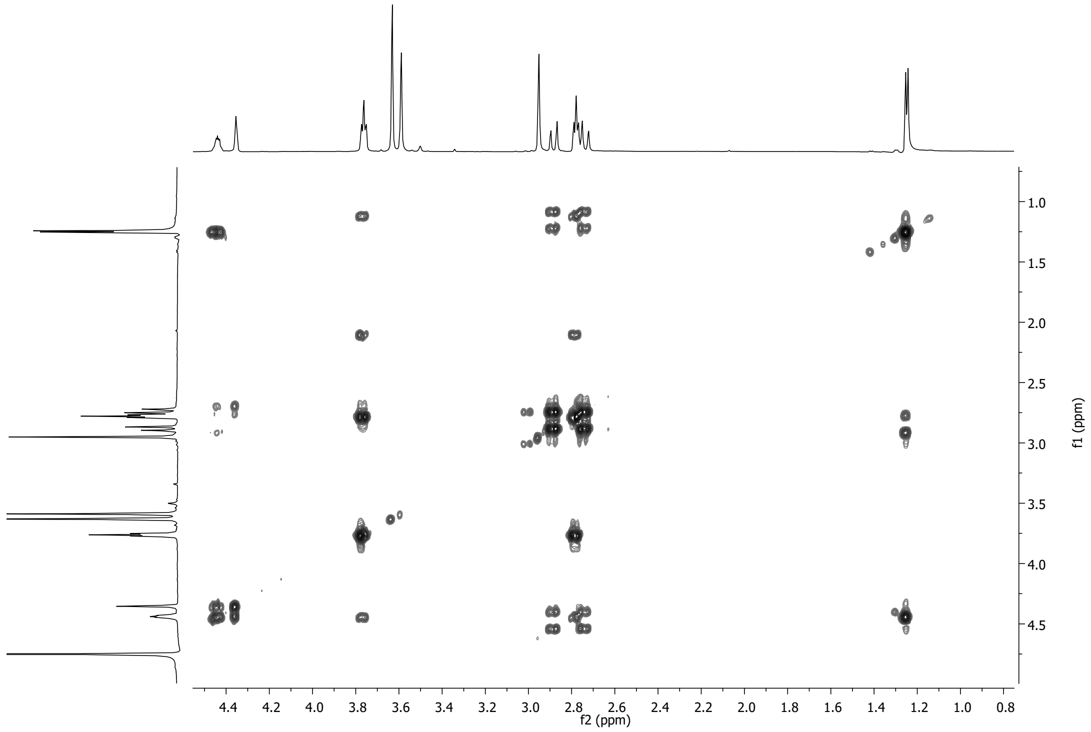

Figure S41. COSY spectrum of compound **6** in D_2O

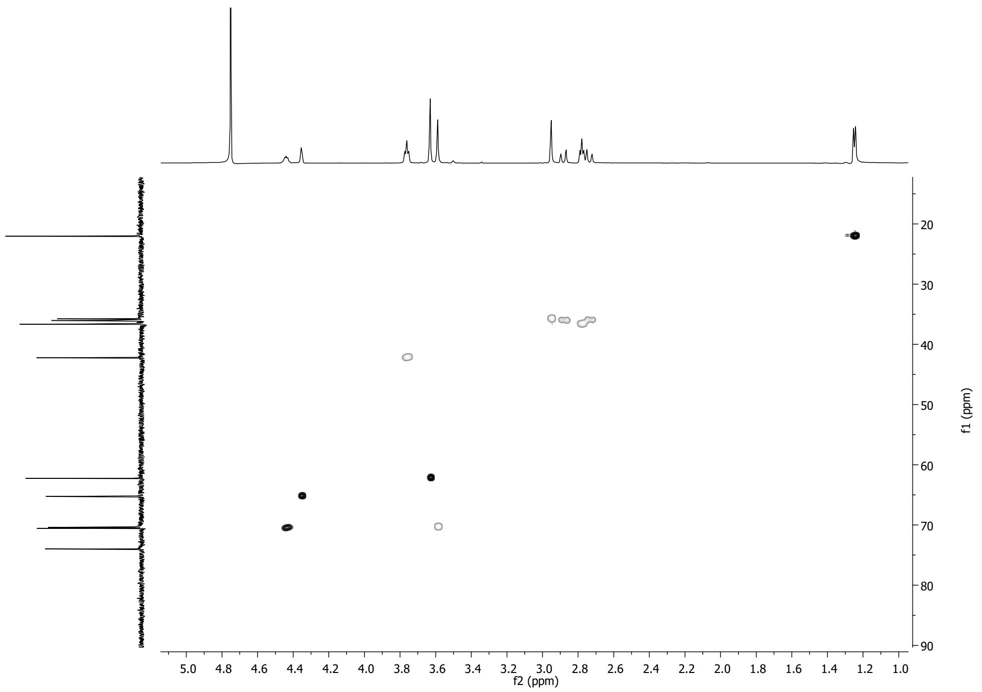

Figure S42. HSQC spectrum of compound **6** in D_2O

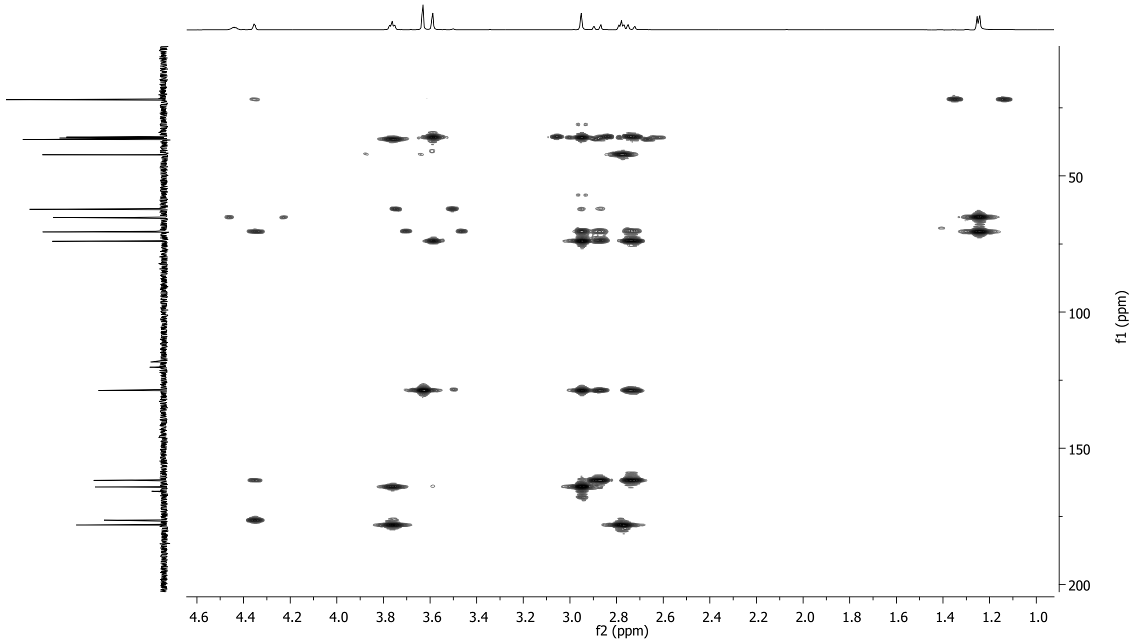

Figure S43. HMBC spectrum of compound **6** in D_2O

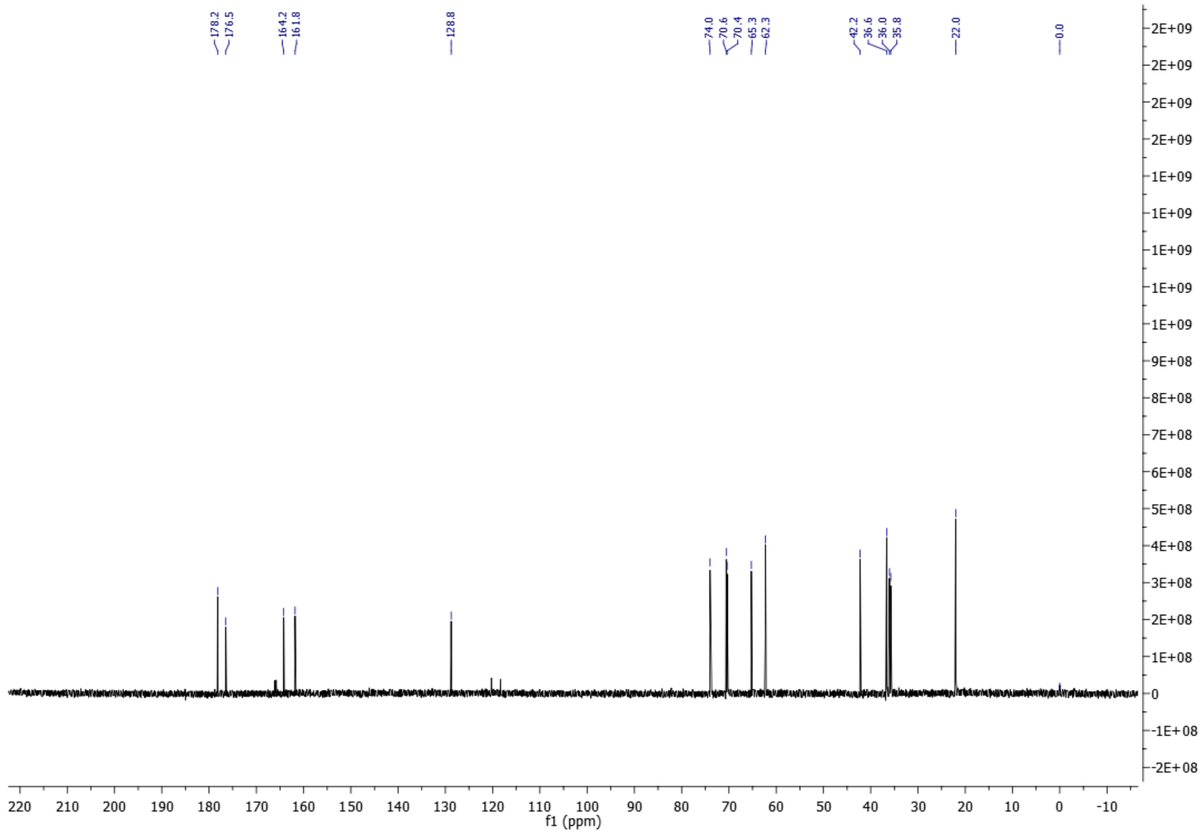

Figure S44. ^{13}C NMR spectrum of compound **6** in D_2O

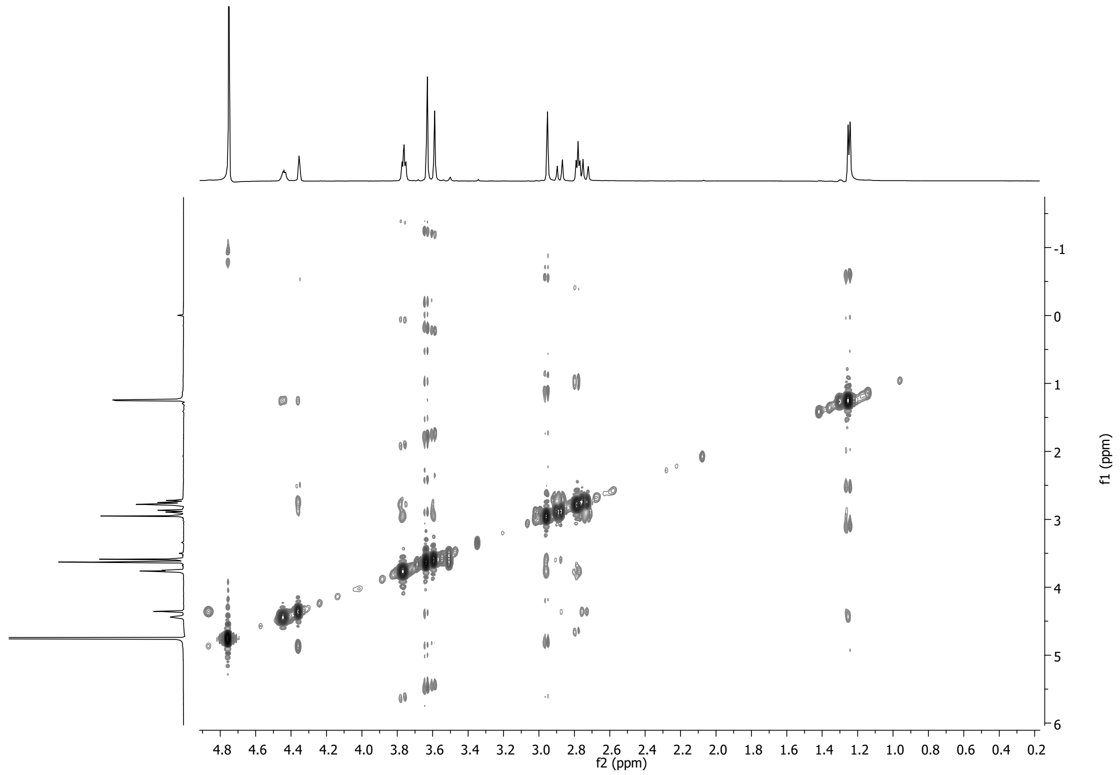

Figure S45. NOESY spectrum of compound **6** in D_2O

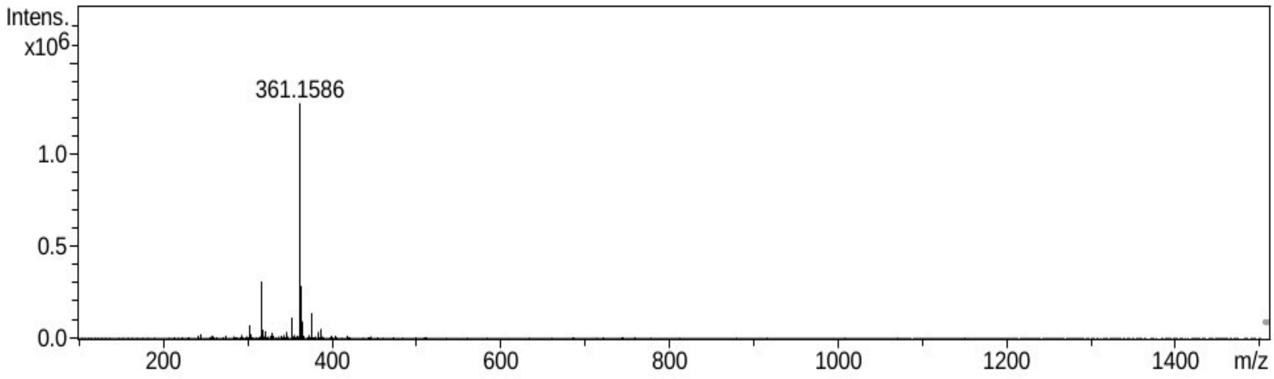

Figure S46. High-resolution mass spectrum of compound **6**

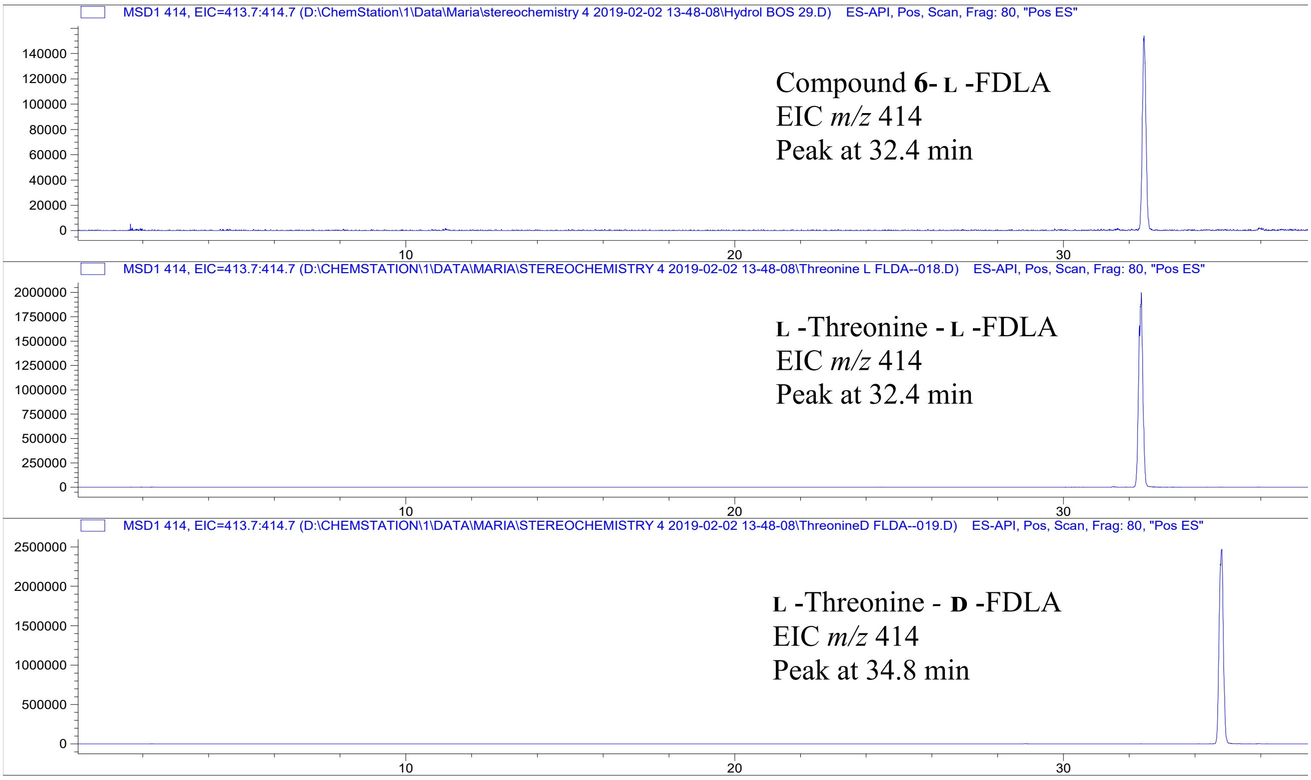

Figure S47. LC-MS of Marfey´s analysis of compound **6**

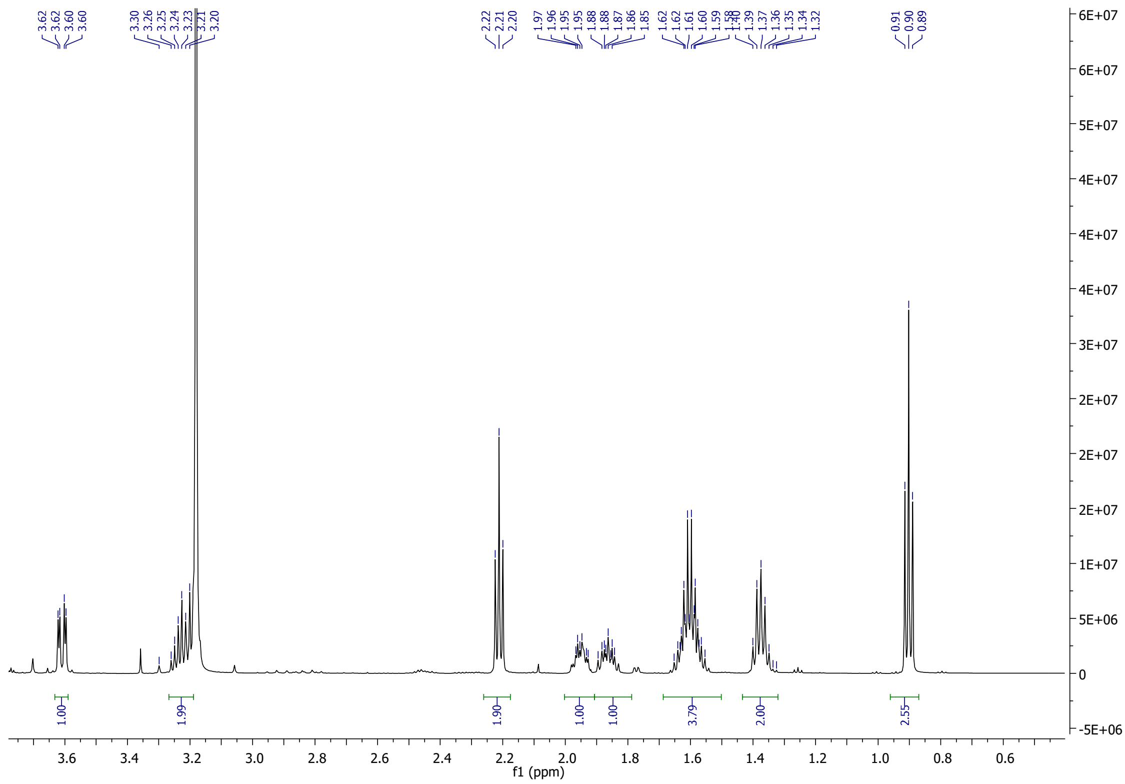

Figure S48. ^{1}H NMR spectrum of compound **8** in D_2O

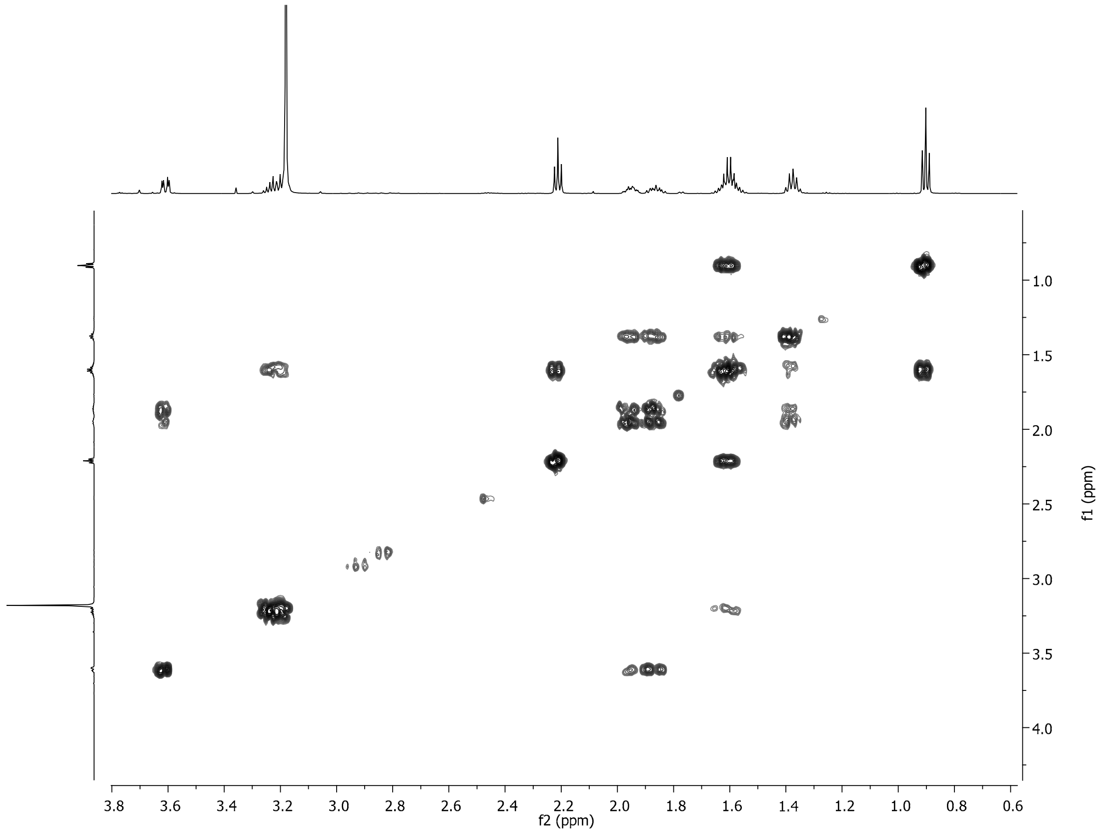

Figure S49. COSY spectrum of compound **8** in D_2O

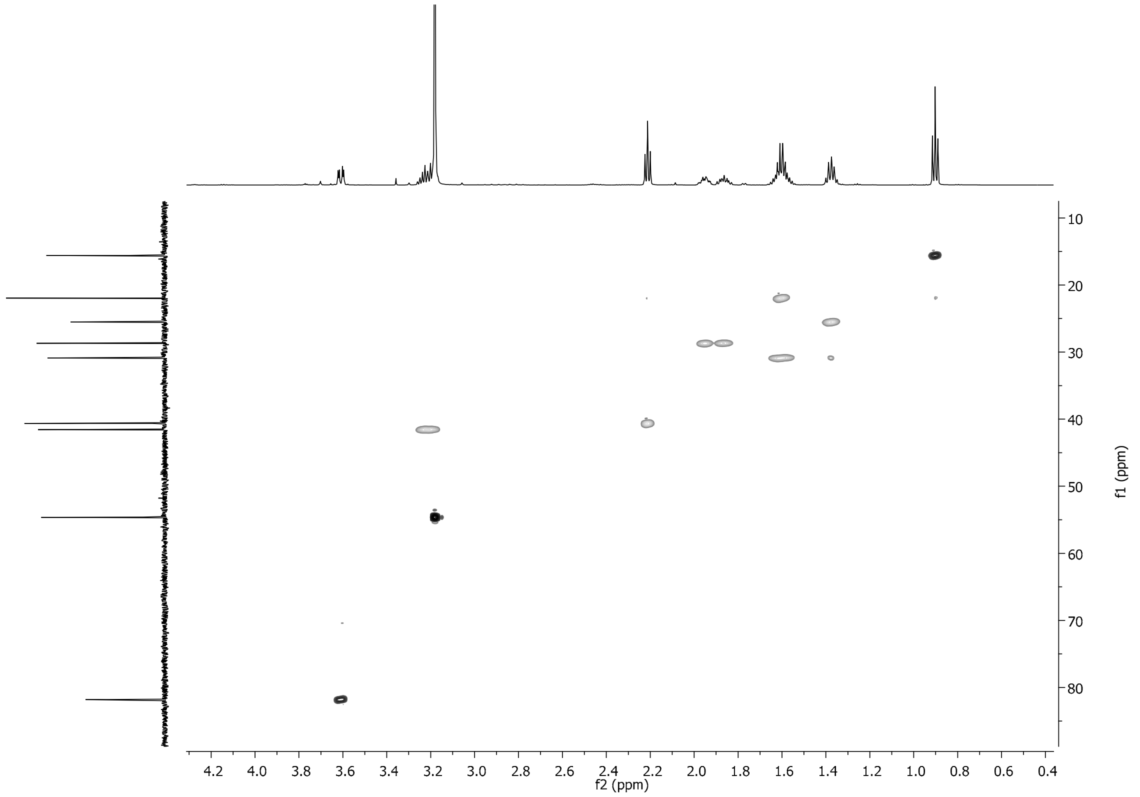

Figure S50. HSQC spectrum of compound **8** in D_2O

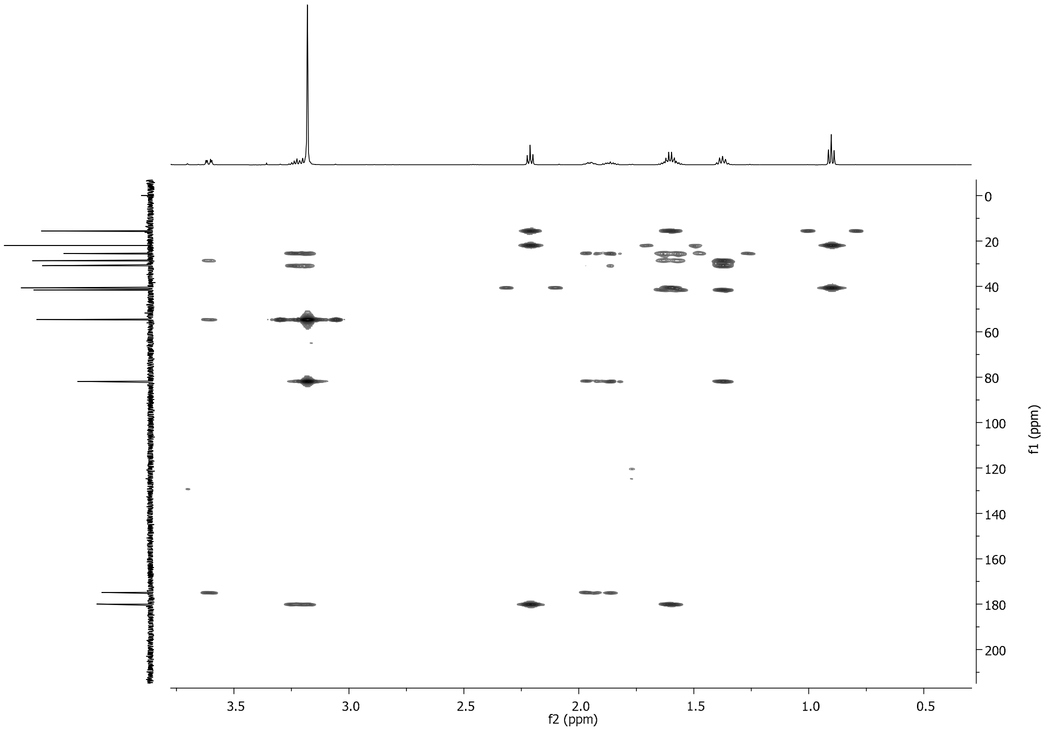

Figure S51. HMBC spectrum of compound **8** in D_2O

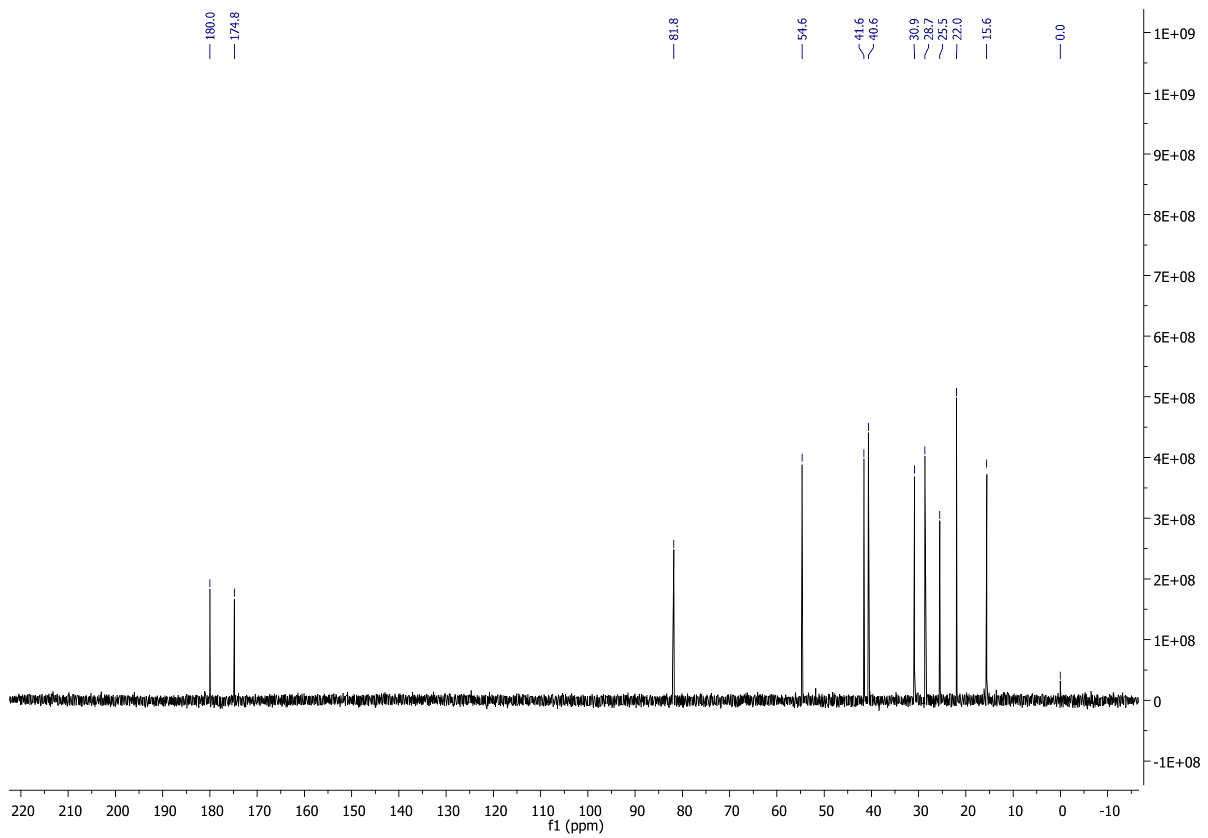

Figure S52. ^{13}C NMR spectrum of compound **8** in D_2O

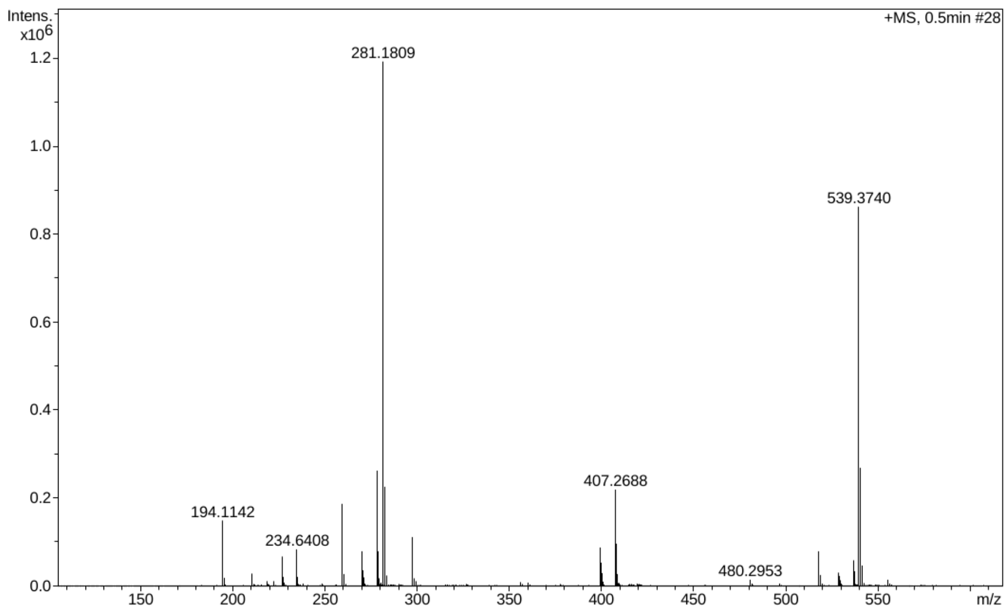

Figure S53. High-resolution mass spectrum of compound **8**
